# Visualisation of exhaled breath metabolites reveals distinct diagnostic signatures for acute cardiorespiratory breathlessness

**DOI:** 10.1126/scitranslmed.abl5849

**Published:** 2022-11-16

**Authors:** Wadah Ibrahim, Michael J. Wilde, Rebecca L. Cordell, Matthew Richardson, Dahlia Salman, Robert C. Free, Bo Zhao, Amisha Singapuri, Beverley Hargadon, Erol A. Gaillard, Toru Suzuki, Leong L. Ng, Tim Coats, Paul Thomas, Paul S. Monks, Christopher E. Brightling, Neil J. Greening, Salman Siddiqui, consortium on behalf of the EMBER consortium

**Affiliations:** 1 Department of Respiratory Sciences, University of Leicester, Leicester, LE1 7RH UK; 2 Institute for Lung Health, NIHR Leicester Biomedical Research Centre (Respiratory theme), Glenfield Hospital, Groby Road, Leicester LE3 9QP; 3 School of Chemistry, University of Leicester, Leicester, LE1 7RH UK; 4 School of Geography, Earth and Environmental Sciences, University of Plymouth, Plymouth, PL4 8AA, UK; 5 Department of Chemistry, Loughborough University, Loughborough, LE11 3TT UK; 6 Leverhulme Centre for Demographic Science, University of Oxford, Oxford, OX1 1JD United Kingdom; 7 Nuffield College, University of Oxford, Oxford, OX1 1NF United Kingdom; 8 Department of Cardiovascular Sciences, University of Leicester, Cardiovascular Research Centre, Glenfield General Hospital, Leicester, LE3 9QP UK; 9 Leicester NIHR Biomedical Research Centre (Cardiovascular theme), Glenfield Hospital, Groby Road, Leicester LE3 9QP; 10 The Institute of Medical Science, The University of Tokyo Shirokane-dai, Minato-ku 4-6-1, 108-8639 Tokyo, Japan; 11 Emergency Medicine Academic Group, Department of Cardiovascular Sciences, University of Leicester, University Road, Leicester LE1 7RH, UK; 12 National Heart and Lung Institute, Imperial College, London, SW3 6LY UK

## Abstract

Acute cardiorespiratory breathlessness accounts for 1 in 8 of all emergency hospitalisations. Early, non-invasive diagnostic testing is a clinical priority that allows rapid triage and treatment. Here, we sought to discover and replicate diagnostic breath volatile organic compound (VOC) biomarkers of acute cardiorespiratory disease and understand breath metabolite network enrichment in acute disease, with a view to gaining mechanistic insight of breath biochemical derangements. We collected and analysed exhaled breath samples from 277 participants presenting with acute cardiorespiratory exacerbations and aged matched healthy volunteers. Topological data analysis (TDA) phenotypes differentiated acute disease from health and acute cardiorespiratory exacerbation subtypes [acute heart failure, acute asthma, acute Chronic Obstructive Pulmonary Disease (COPD) and community-acquired pneumonia]. A multi-biomarker score (101 breath biomarkers) demonstrated good diagnostic sensitivity and specificity (≥ 80%) in both discovery and replication sets and was associated with all-cause mortality at 2 years. In addition, VOC biomarker scores differentiated metabolic subgroups of cardiorespiratory exacerbation. Louvain clustering of VOCs coupled with metabolite enrichment and similarity assessment revealed highly specific enrichment patterns in all acute disease subgroups, for example selective enrichment of correlated C_5-7_ hydrocarbons and C_3-5_ carbonyls in heart failure and selective depletion of correlated aldehydes in acute asthma. This study identified breath VOCs that differentiate acute cardiorespiratory exacerbations and associated subtypes and metabolic clusters of disease-associated VOCs.

## 1 Introduction

Breathlessness due to cardiorespiratory diseases accounts for more than 1 in 8 of all emergency admissions to hospital (1). Despite the same presenting symptom, the aetiology of acute breathlessness is highly varied, with diverse disease trajectories and therapeutic options. Diagnostic evaluation of acute breathlessness is heavily reliant on investigations such as blood-based biomarkers [e.g. C-reactive protein (CRP), B-type natriuretic peptide] and radiological procedures. These biomarkers have clinical utility primarily in patients with single pathologies, but have poor discriminatory power in patients with multifactorial presentations of acute breathlessness and are particularly challenging to interpret in the context of pre-admission treatment exposure (e.g. antibiotics for pneumonia and admission CRP values) (2).

Breathomics, the characterisation of volatile organic compounds (VOCs) in exhaled breath, enables the evaluation of diagnostic and prognostic biomarkers in acute breathlessness, directly from the lung as well as incorporating metabolites from the systemic circulation (3). The assessment of exhaled, low-molecular weight biochemicals, chemically classified as VOCs, has been presented as a new paradigm for the development of rapid, non-invasive diagnostic and prognostic biomarkers. However, the scarcity of robustly powered clinical studies, combined with a lack of standardisation in sample collection and analysis as well as data and chemometric processing, have delayed further translation of this technology to clinical settings.

Notwithstanding these challenges, the potential of breathomics is becoming increasingly recognised in research and therapeutic development in respiratory diseases. The emergence of powerful high-resolution mass spectrometry and multidimensional separation technologies such as comprehensive two-dimensional gas chromatography coupled with mass spectrometry (GCxGC-MS), which provides visual readouts of breath-based biomarkers (4, 5), has facilitated research advances. Although chemometric analyses play a vital role in this field, the enhanced dimensionality of GCxGC-MS data enriches established chemometric and imaging-based characterisation methods for visualising, extracting and quantifying VOC markers from complex and previously unresolved matrices.

Herein, we present a real-world, prospective study of acutely unwell hospitalised patients presenting with breathlessness due to severe exacerbations of cardiorespiratory aetiology (asthma, COPD, heart failure or pneumonia) and healthy controls. By isolating and visualizing exhaled VOCs with GCxGC-MS, coupled with rigorous clinical phenotyping, exhaled breath metabolites were shown to have high diagnostic accuracy for severe cardiorespiratory exacerbations (including in the presence of diagnostic uncertainty) and to be dysregulated across several pertinent volatile classes in different clinical subtypes of cardiorespiratory exacerbation. This research provides pivotal evidence that shows how breath biomarker platforms may be used in acute care and demonstrates the potential for translation of this technology into a real-world clinical setting.

## 2 Results

### 2.1 Participant demographics and clinical characteristics

**Fig. 1:**
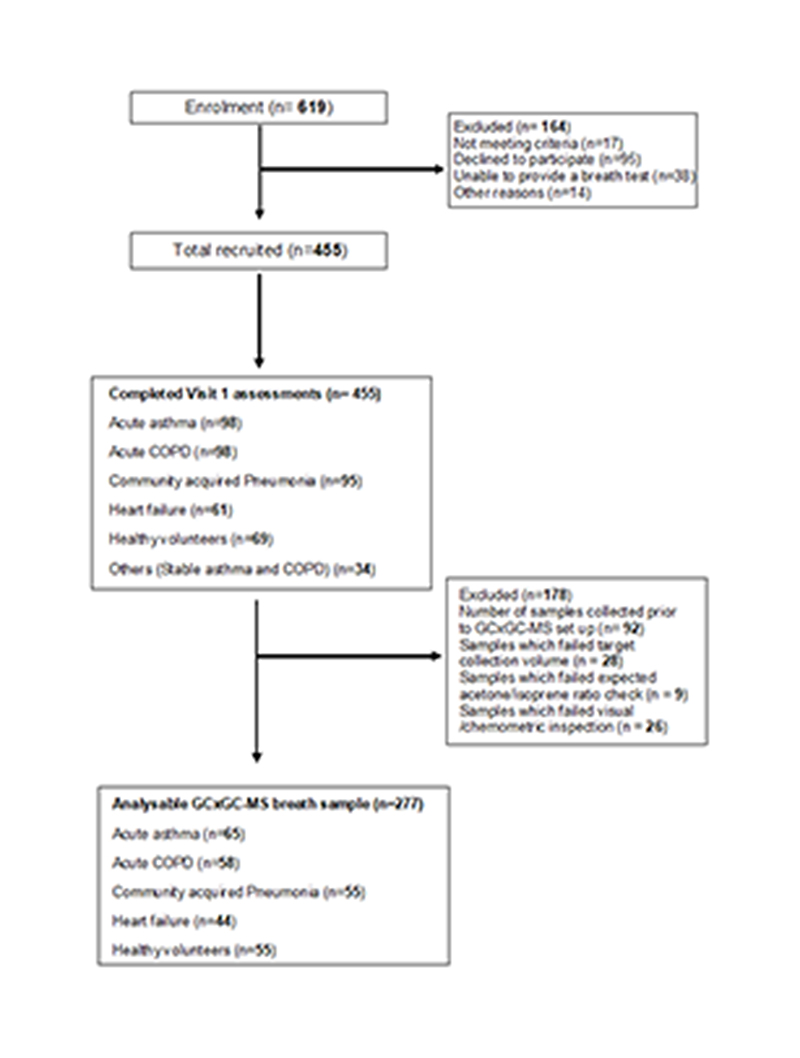
Study Consort diagram. Consort diagram outlining the acute study recruitment and number of analysable GCxGC-MS breath samples.

As part of the East Midlands Breathomics Pathology Node (EMBER), exhaled breath from 277 participants recruited from acutely breathless hospitalised patients and matched healthy controls was sampled (Figure 1). **1).** Sample size calculations are detailed in (Methods section ‘sample size estimation’ and Table S1). Breath samples were analysed to identify dysregulation of metabolic classes in cardiorespiratory disease and investigate whether exhaled VOC profiles could predict acute cardio respiratory exacerbations despite diagnostic uncertainty, and thus have a potential role in phenotyping acute cardiorespiratory breathlessness (fig. S1). Participants’ mean (SD) age was 60.8 ± (16.8) years, 51% were males, 30 patients required supplemental oxygen on admission and the mean admission modified early warning score (mEWS-2 score) was 2. The cohort was made up of patients presenting with the following exacerbation subtypes: acute severe asthma (n= 65), acute severe COPD (n= 58), acute severe heart failure (n=44), community acquired pneumonia (n=55), and healthy volunteers (n=55), recruited between May 2017 and December 2018. Participants’ demographic and clinical characteristics are summarised in (Table 1). Breath samples were collected using a ReCIVA device, adopting a standardised sampling and gated protocol that enriches alveolar volatiles (6), and analysed using thermal desorption (TD) coupled to comprehensive two-dimensional gas chromatography (GCxGC) with dual flame ionisation detection (FID) and mass spectrometry (MS).

### 2.2 Unbiased discovery using TDA identifies breath markers of acute disease

Topological data analysis is an unsupervised machine-learning tool used for the analysis of large-scale, high-dimensional, complex datasets. It is highly sensitive to patterns that are often overlooked by other data reduction tools like Principal Component Analysis (PCA) (30).

TDA is a well-established data analytic technique for unbiased data driven discovery based phenotyping (30). TDA has proven to be a powerful tool, yielding critical insights in the prognostic phenotyping (31), cancer imaging biomarker stratification (32), disease classification using pathology biomarkers (33), omics based cancer phenotyping (34). Several publications have reported the use of TDA in the metabolomics field, for example, unbiased lipid phenotyping of lung epithelial lining fluid (35).

To achieve an unbiased discovery of exhaled VOCs predictive of the acute disease groups, patients were block randomised *post-hoc* into a discovery cohort of 139 participants (acute asthma n= 33, acute COPD n= 29, acute heart failure n=22, community acquired pneumonia n=28, healthy volunteers n=27), and a replication cohort of 138 participants (acute asthma n= 32, acute COPD n= 29, acute heart failure n=22, community acquired pneumonia n=27, healthy volunteers n=28). Randomisation allowed internal replication of diagnostic breath biomarkers, whilst adjusting for relevant confounders. Details of the randomisation and further clinical characteristics of the cohorts can be found in (tables S2-S3). Chemometric analysis and quantification of VOCs was performed blinded to clinical diagnosis by two analytical chemists (MW and RC), with biostatistical analyses linking subject identifier to chemometric biomarkers performed following data lock by an independent statistician (MR).

**Fig. 2:**
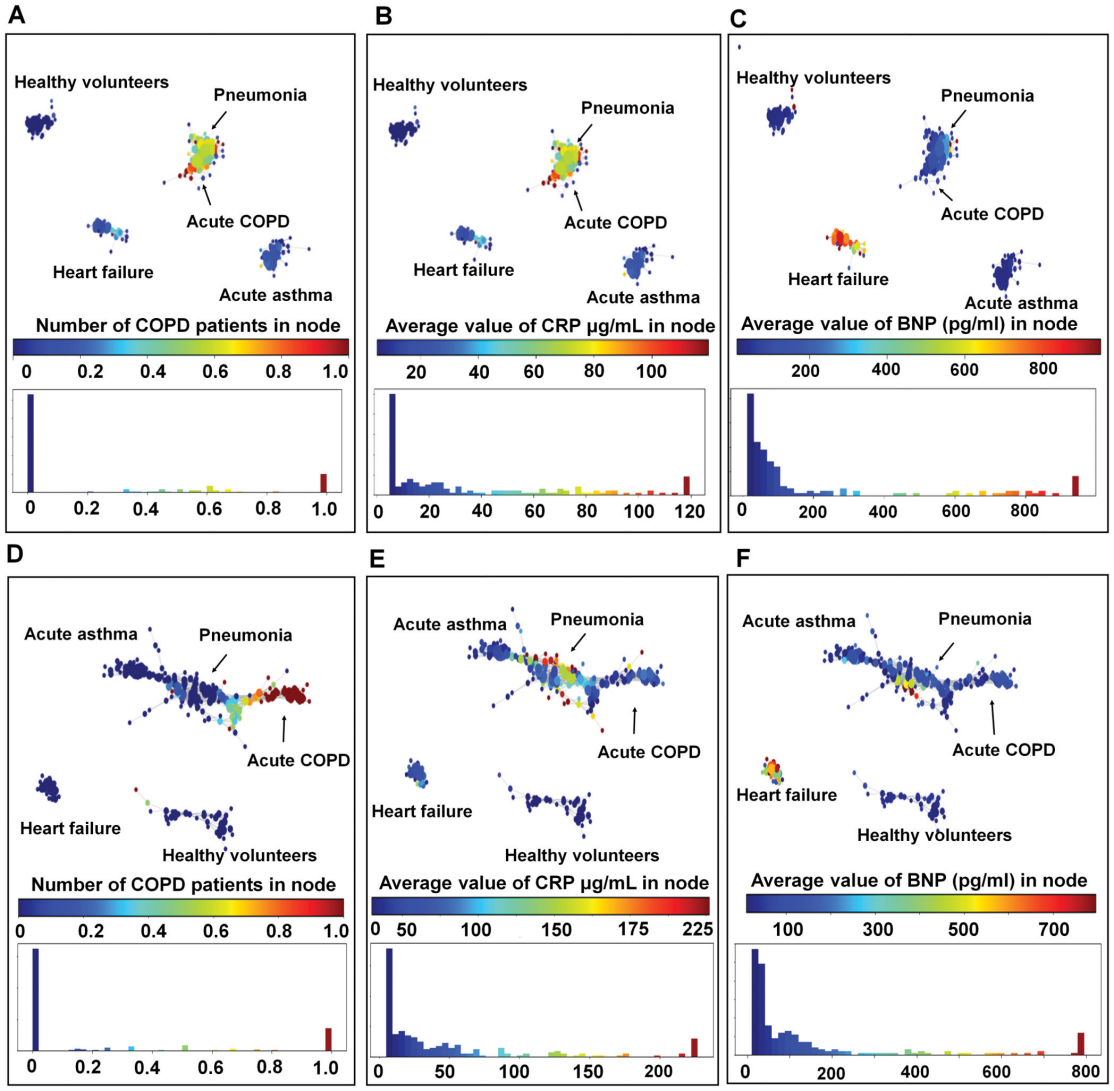
Topological data analysis (TDA) representing the various acute disease groups annotated by blood biomarkers. Each circle or ‘node’ in the TDA graph represents a subject or group of subjects. Similar subjects are grouped together in the same node and the relative similarity of the subjects is represented by the proximity of the nodes. The size of each node is determined by the number of subjects within it. **A:** Visual mapping of the acute disease groups in the discovery cohort (n=139), based on the discriminatory 805 features and coloured by proportion of acute COPD exacerbations in each node. **B:** The network is colour coded by the average values of CRP in each node in the discovery cohort (n=139). Higher CRP values corresponded topologically with the COPD and pneumonia patients. **C:** The network is colour coded by the average values of BNP in each node in the discovery cohort (n=139). Higher BNP values corresponded topologically with the heart failure patients. **D:** The network is coloured by proportion of acute COPD exacerbations in each node in the replication cohort (n=138). In replication cohort, Pneumonia and COPD exacerbation subjects occupied polar ends of the same TDA network. **E:** The networks are coloured by the average values of CRP in each node. High CRP values corresponded topologically with the pneumonia subjects. **F:** The networks are coloured by the average values of BNP in each node. High BNP values corresponded topologically with the heart failure subjects.

805 unique chromatographic features (peaks) were detected across the breath sample set using TD-GCxGC-FID/MS, with 404 features detected on average in each sample. Topological data analysis (TDA) applied to these 805 chromatographic features yielded topologically distinct networks that distinguished underlying causes of acute breathlessness whilst anchoring to corresponding blood-based biomarkers in both the discovery and replication cohorts (Figure 2). Specifically, healthy volunteers and patients with acute heart failure formed distinct topological groupings in both discovery and replication populations. Respiratory admissions due to acute asthma, acute COPD and pneumonia formed a topological continuum albeit within distinct regions of a single network in the replication cohort; similar findings were observed in the discovery cohort, with the exception of acute asthma forming a distinct grouping.

### 2.3 Breath biomarker clinical prediction scores

To create a concatenated list of exhaled breath biomarkers suitable for diagnostic application, we applied a threshold of 80% feature-presence per patient group, below which features were removed to effectively reduce the number of features used in subsequent models with more than 20% of zero values for peak areas (fig. S2). We found that the zero-valued peak areas were randomly distributed across the disease groups in all but seven features. The exclusion of the seven features where there was some evidence that zero-valued peak areas were not randomly distributed across the disease groups did not alter the results of the regression models.

Further filtering steps using least absolute shrinkage and selection operator (LASSO) and elastic net regression methods, followed by removal of 38 peaks that were considered to be chemical and material artefacts (e.g. siloxanes), generated a final panel of 101 exhaled breath volatiles (tables S4-S8). Therefore, the analysis plan permitted the identification of a rich and chemically diverse response in the VOC profile as opposed to only a handful of individual VOC markers and afforded the generation of biomarker scores. The data was examined for batch effects and was adjusted accordingly. Batch effects detected related to major instrument maintenance events, which occurred twice creating three groups. No contributions were observed based on the ReCIVA device used, operator, time of day, or volume of breath sample collected, most likely nullified by the simultaneous and consecutive recruitment across all cohorts throughout the study to reduce potential biases (fig. S3-4).

**Fig. 3:**
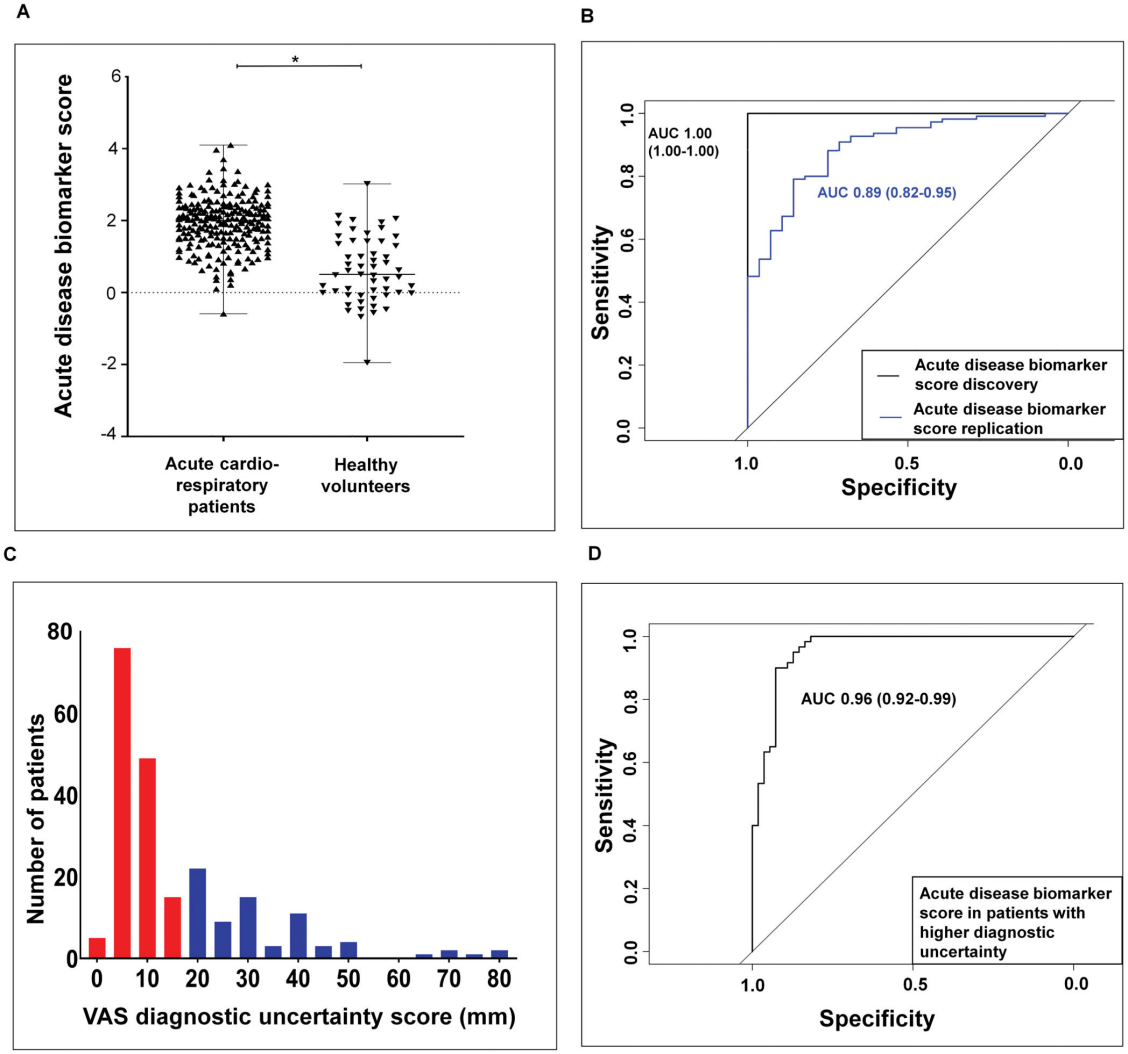
Diagnostic accuracy of an acute VOC biomarker score. **A** Scatter plot demonstrating significant difference between breath VOC biomarker score values in acute cardiorespiratory patients compared to healthy volunteers. The black horizontal line within the scatter plot represents the median value of the biomarker score. Mann Whitney test *P < 0.0001. **B:** Receiver operating characteristic (ROC) curve of participants in the discovery [black line - AUC 1.00 (1.00-1.00)] and replication [blue line - AUC 0.89 (0.82-0.95)] cohorts *P* < 0.0001. **C:** Histogram showing the number of patients with higher diagnostic uncertainty (blue bars with values > upper quartile value of 20 mm). **D**: ROC curve assessing the discriminatory power of exhaled breath VOCs in participants with higher diagnostic uncertainty. AUC 0.96 (0.92-.99) *P* < 0.0001

The value of the generated acute disease VOC biomarker score was found to be higher in acute cardiorespiratory patients compared to healthy volunteers (Figure 3A). For the discovery cohort (n=139), the acute disease VOC biomarker score effectively differentiated participants with acute cardiorespiratory exacerbations from age-matched healthy controls with an area under the curve (AUC) of 1.00 (1.00-1.00) *P* < 0.0001, sensitivity 1.00 (1.00-1.00), specificity (1.00-1.00), positive predictive value (PPV) 1.00 (1.00-1.00), negative predictive value (NPV) (1.00-1.00). For the replication cohort (n=138), the same VOC biomarker score differentiated participants with acute disease from healthy controls with AUC 0.90 (0.83-0.96) *P*<0.0001, sensitivity 0.88 (0.82-0.94), specificity 0.79 (0.63-0.94), PPV of 0.95 (0.91-0.99), NPV of 0.51 (0.36-0.65) (Figure 3B).

To evaluate the impact of potential confounders on our model classification, we re-ran our statistical models, adjusting for the following factors: (i) smoking status (current, ex-smoker or never smoker); (ii) time between hospital admission and the acquisition of the breath samples, as this time period is often the period within which acute treatments are delivered; (iii) the modified early warning score 2 (mEWS-2), which is a composite acuity score combining respiratory rate, oxygen saturations, systolic blood pressure, heart rate, degree of consciousness, confusion and body temperature for each patient; and (iv) prior exposure to either antibiotics or steroids for cardiorespiratory illness in the fortnight prior to the index admission. We observed improved diagnostic accuracy in the replication cohort [AUC 1.00 (1.00-1.00), *P* <0.0001] when considering these adjustments, which would be expected with the inclusion of acuity markers for the classification of acute illness.

Following a clinical adjudication process (Methods: section ‘clinical adjudication’), each patient was assigned a degree of clinical diagnostic uncertainty using a 100-mm visual analogue scale (VAS) at the point of clinical triage (Figure 3C). Diagnostic uncertainty was defined as patients with values higher than or equal to the upper quartile of 20 mm on the VAS. The acute disease VOC biomarker score was able to identify acute disease with an AUC 0.96 (0.92-0.99) *P* <0.0001, sensitivity 0.90 (0.82-0.97), specificity 0.92 (0.85-0.99), PPV 0.93 (0.86-0.99), NPV 0.89 (0.81-0.97) (Figure 3D).

### 2.4 Exhaled breath biomarker disease-specific scores correlate with blood-based biomarkers and admission observations

**Fig. 4:**
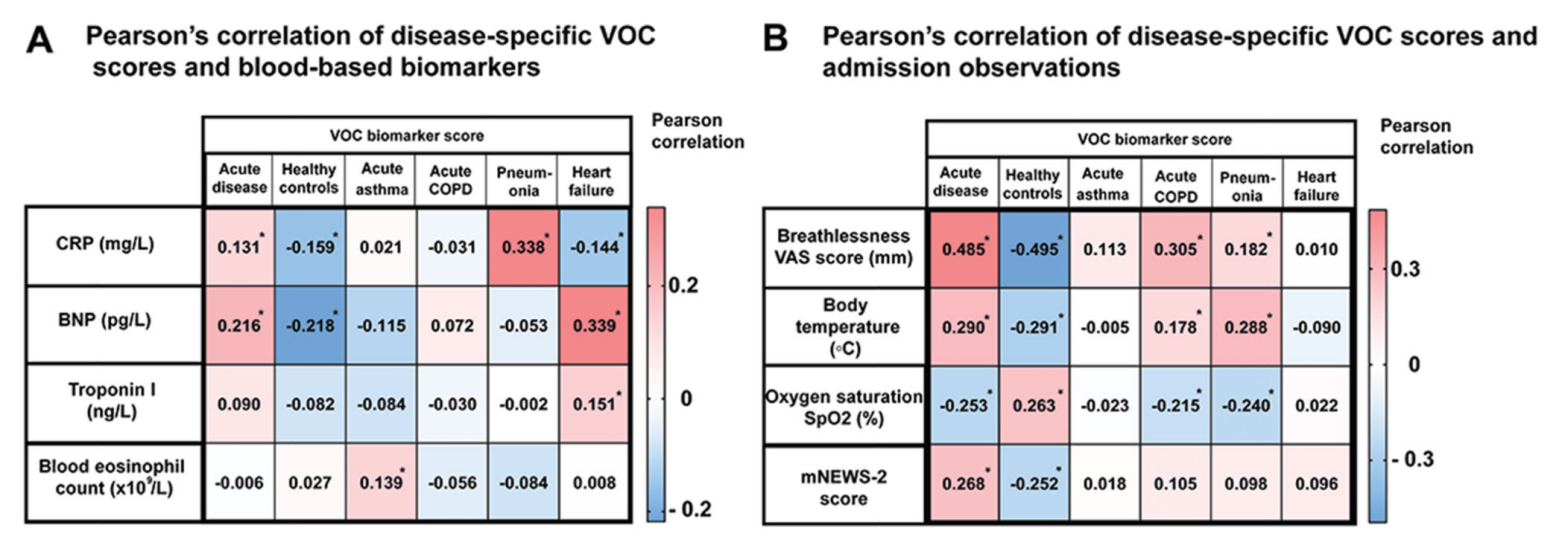
Correlation of VOC biomarker score with blood biomarkers and disease acuity. **A:** Pearson’s correlation of disease-specific VOC scores and blood-based biomarkers. Pearson correlation demonstrating the positive and negative correlations between breath VOC scores and blood-based biomarkers. *P < 0.05. **B:** Pearson’s correlation of disease-specific VOC scores and admission observations. Pearson correlation between the VOC biomarker score and admission vital signs. VAS: Visual Analogue Scale (100 mm), participants were asked to rate their breathlessness on a 100 mm VAS on admission.

As previously described, VOC biomarker scores were generated for each of the acute disease subgroups and healthy subjects without cardiorespiratory breathlessness. There was a weak but positive correlation in the combined discovery and replication cohorts (n=277) between the VOC subgroup scores for pneumonia and CRP (n=277, r=0.33, *P* <0.0001) and acute heart failure and Brain Natriuretic Peptide (BNP) (n=277, r=0.33, *P* <0.0001), in addition to a negative correlation between the healthy-state VOC score and CRP and BNP (n=277, r= -0.15, *P* <0.0001, and -0.21, *P* <0.0001 respectively) (Figure 4A). Correlations were also identified between the acute disease VOC score and vital observations carried out during triage (Figure 4B).

The acute disease VOC score was also associated with 2-year all-cause mortality, but not with the risk of 60-day readmission (fig. S5).

### 2.5 Diagnostic accuracy of breath biomarker scores in cardiorespiratory disease subgroups

A multinomial regression model using elastic net regularization was fitted to the matrix of 101 breath biomarkers with the 10-fold cross validation repeated 1,000 times. Linear combinations of the most stable features from the multinomial regression model fitted to the 101 biomarkers formed a set of scores for predicting probability of belonging to the different disease groups (acute asthma, acute COPD, pneumonia, heart failure or healthy volunteers).

The overall classification accuracy for the statistical model generated from 101 breath biomarkers was assessed by comparing the balanced accuracy of model trained using the true class labels versus the balanced accuracy of the same model tested using randomly shuffled class labels. This process was repeated 1,000 times. The balanced accuracy is reported in (fig. S6A) the acute disease biomarker score in the discovery cohort, (fig. S6B) the acute disease biomarker score in the replication cohort and (fig. S6C) the multinomial biomarker scores for the five subgroups acute asthma, acute COPD, heart failure, pneumonia and healthy volunteers. NB: replication was not evaluated in the subgroups as the study was not powered to do this.

For the pooled cohort (*n* = 277), the overall classification accuracy using all five biomarker scores was 0.72, 95% CI (0.67 - 0.77). The balanced accuracy for acute asthma was 0.83, for acute COPD 0.78, for heart failure 0.80, for community acquired pneumonia 0.79, and for healthy controls was 0.93 (fig S5).

Further comparative ROC analyses were performed based upon the observed separation of asthma from pneumonia/COPD acute groups, and heart failure from other acute exacerbation groups in the discovery and replication TDA analyses. The diagnostic AUC accuracy of the asthma biomarker score against pooled Pneumonia and COPD cohorts was AUC: 0.70 (0.62-0.78) *P* <0.0001, sensitivity 0.72 (0.64-0.83), specificity 0.64 (0.55-0.73), positive predictive value (PPV) 0.54 (0.43-0.64), negative predictive value (NPV) 0.80 (0.72-0.88). Receiver operating curve (ROC) analysis to assess the diagnostic value of the heart failure biomarker score against other acute disease groups was AUC: 0.78 (0.70-0.86) *P* <0.0001, sensitivity 0.77 (0.64-0.89), specificity 0.71 (0.64-0.78), PPV 0.40 (0.29-0.50), NPV 0.92 (0.88-0.97) (fig. S7).

The median values of the exhaled breath VOC scores and their distribution across disease subgroups are detailed in (fig. S8). Figure S9 is a Venn diagram demonstrating the distribution of the final panel of 101 exhaled breath biomarkers across the different disease groups.

We also ran our models adjusting for the following factors: (i) smoking status (current, ex-smoker or never smoker; (ii) time between hospital admission and the acquisition of the breath samples, as this time period is often the period within which acute treatments are delivered; (iii) the modified early warning score 2 (mEWS-2), which is a composite acuity score combining respiratory rate, oxygen saturations, systolic blood pressure, heart rate, level of consciousness and confusion for each patient; and (iv) prior exposure to either antibiotics or steroids for cardiorespiratory illness in the fortnight prior to the index admission. We observed only marginally improved diagnostic accuracy; acute asthma - AUC 0.88 (0.831,0.933), *P* <0.0001, COPD - AUC 0.86, (0.808,0.918), *P* <0.0001, heart failure - AUC 0.91 (0.849,0.969) *P* <0.0001, community acquired pneumonia – AUC 0.91 (0.863,0.953), *P* <0.0001, and healthy controls AUC 1.0, suggesting limited confounding influence of disease acuity on our biomarker scores (Auxiliary supp table 1). Replication was not performed in the subgroups, as the EMBER study was not powered for disease subgroup diagnostic accuracy.

### 2.6 Chemical classification of predictive markers in disease groups

Chemical identification of the 101-biomarker panel involved comparison with an authentic reference compound in accordance with the Metabolomics Standard Initiative (MSI) Level 1 criteria for metabolite identification. The most common chemical classes associated with acute breathlessness in this study included straight-chain and methyl-branched hydrocarbons (30%), ketones (10%), aldehydes (8%) and terpenes (13%), followed by sulphur-containing VOCs (7%), alcohols (6%), aromatics (5%), esters (3%), nitrogen-containing VOCs (3%), ethers (2%), halogen-compounds(1%), and an assortment of other less prevalent and less relevant classes such as acrylates (12%) (Table S9).

### 2.7 Metabolite set enrichment and chemical similarity analysis

Unlike functional indications, which are reliant on mapping metabolites with known, well-annotated metabolic pathways, metabolic changes indicative of response can be derived independently. To derive clues of responsive indication, the panel of 101 features was assessed for co-varying clusters (i.e. metabolite sets).

**Fig. 5:**
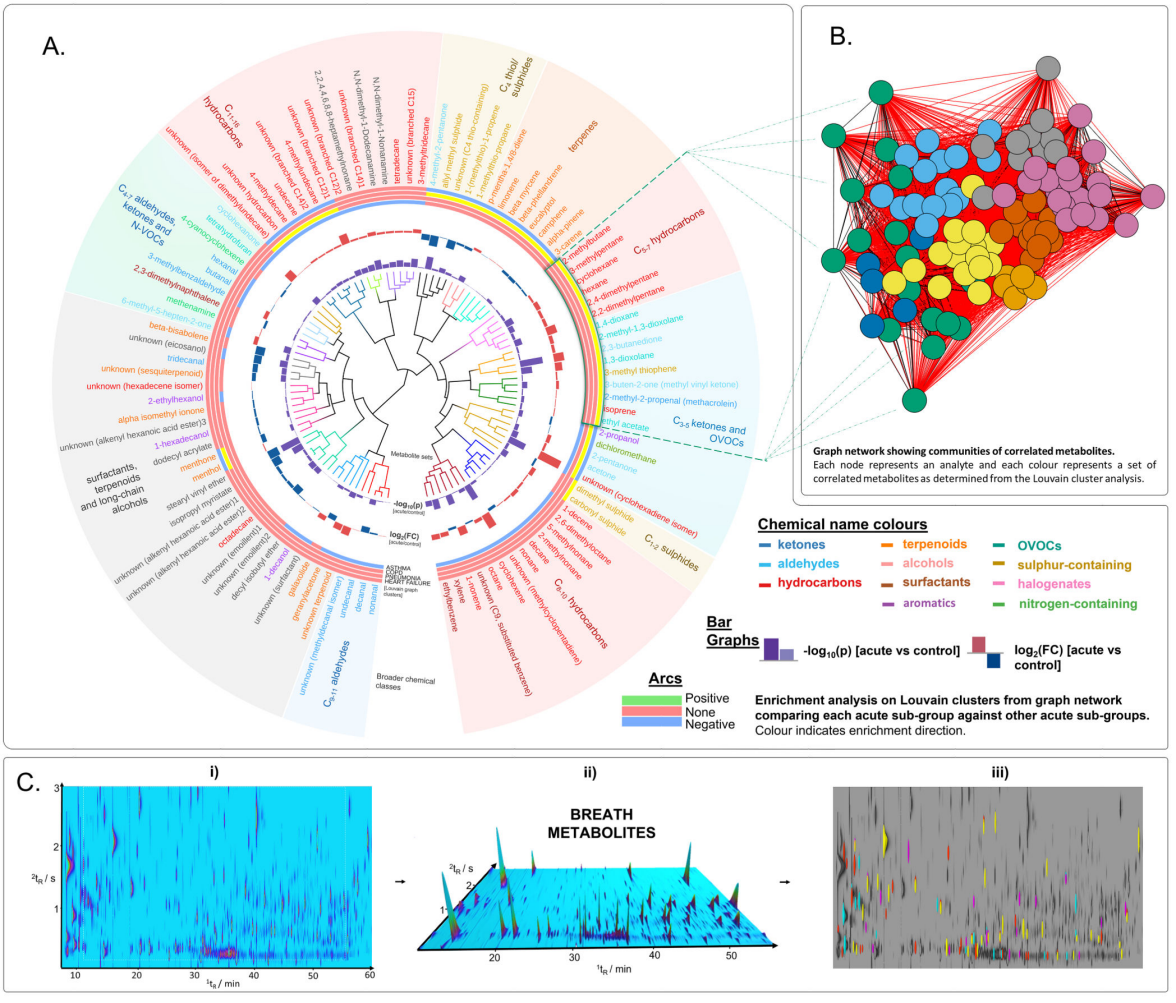
VOC biomarker chemical enrichment in acute cardiorespiratory exacerbations. **A:** Circular correlation tree generated based on metabolite set enrichment and chemical similarity analysis of 101 breath volatiles associated with acute breathlessness. Branches depict metabolite sets derived using the ChemRICH; bar graphs portray -log10(p) and log2(fold change) values of 101 features extracted using LASSO regression (table S4) in acute breathlessness compared with control group. The arcs represent the Louvain clusters, derived from the correlation graph (green for upregulated, red for not significant, blue for downregulated according to K-S test result). Chemical names are coloured based on their chemical classification and coloured regions used to summarise broader chemical groups. **B:** Correlation graph showing metabolite communities identified using Louvain clustering, with the identity and location of the cluster enriched in heart failure projected onto the circular dendrogram. **C:** i) Example GCxGC chromatogram showing complex profile of breath metabolites; ii) 3D render of chromatogram showing visualisation of breath markers; and iii) phenotypic differences based on features included in the breath biomarker scores (table S9) (yellow, asthma; red, pneumonia; magenta, COPD; cyan, heart failure).

Metabolite sets were derived based on Ward hierarchical cluster analysis using the ChemRICH method reported previously (7) (Figure 5A & figure S10), and broader communities were derived from Louvain cluster analysis (Figure 5B and tables S10-S13) to help interpret the correlation graphs. Overall, twenty metabolite sets were identified using ChemRICH, eleven of which were enriched during acute cardiorespiratory exacerbations. The seven metabolite sets that were upregulated consisted of predominantly acyclic and branched hydrocarbons (sets 3, 5, 7 and 9 in figure S10).The results from the analysis herein demonstrated enriched, co-expression of hydrocarbons with high chemical similarity providing primary evidence of exhaled VOCs indicative of disease response measured in vivo. This is clearly seen in Figure 5A, with the metabolite sets (inner tree) labelled by broader chemical classifications (outer ring); C_5-7_, C_8-10_ and C_11-16_ form clusters based on carbon number also exhibiting the highest change during acute exacerbation. Owing to the increased separation power afforded by GCxGC-MS, it was possible to map the VOC signatures back to the multidimensional chromatograms for the visualisation of exhaled breath metabolites which revealed distinct diagnostic signatures for acute cardio-respiratory breathlessness (Figure 5C).

## 3 Discussion

In this pragmatic, acute-care study, we evaluated the validity of breath biomarker profiling in high-acuity patients presenting with acute cardiorespiratory breathlessness. Using GCxGC-MS, we observed that robust and validated sampling of alveolar breath coupled with GCxGC-MS biomarker characterisation demonstrated high diagnostic accuracy for acute cardiorespiratory exacerbations.

We have also identified putative biomarker scores from subsets of breath VOC biomarkers that classify cardiorespiratory exacerbation subtypes and warrant validation in appropriately powered replication studies. Furthermore, we have identified several classes of VOCs that are highly correlated and selectively enriched or supressed in acute disease (including subgroups) compared to health, providing potential insights into broad dysregulation of the metabolome in acute cardiorespiratory exacerbations.

The analytical methods described herein were underpinned by robust biomarker development protocols using TD-GCxGC-FID/MS, integral to the standardisation and integration of breath analysis in large translational studies (5, 8). Several potential confounders including batch variation were addressed in detail. Furthermore, biomarker quantification of the 101 VOCs followed the recommendations of the MSI, with 58 compounds identified against pure and traceable standards (level I), 21 putative identities based on mass spectral and retention index library matches (level 2), and 22 classified on mass spectral data (9). Markers that appeared to localise to individual cardiorespiratory conditions could be readily visualised using TDA.

The identification of hydrocarbons and carbonyls as the major chemical classes was consistent with current mechanistic understanding, postulated as chemical endpoints of lipid peroxidation resulting from oxidative stress during inflammation. Aldehydes such as nonanal, decanal and hexanal were predictive for asthma; ketones included 2-pentanone (asthma), cyclohexanone (pneumonia) and 2,3- butanedione (COPD) which were all previously reported (4, 10–14). Individual hydrocarbons such as 2,4- and 2,2-dimethylpentane, 2- methylbutane, 4-methyldecane, 5-methylnonane and isoprene have been previously reported as predictive for pneumonia and heart failure (12, 15). Sulphur-containing VOCs, such as 3-methylthiophene, allyl methyl sulphide and carbonyl sulphide (found to be predictive of COPD) are associated with bacterial metabolism, postulated to originate from the gut (16) and on occasions as a result of radiation injury (17); however, 2,3-butanedione, also predictive of COPD, has been identified as a metabolic product of bacterial isolates from patients with cystic fibrosis (CF) (16) and postulated to be an important metabolite in monitoring lung infection in CF, COPD and pneumonia. We acknowledge that the biological origin of most VOCs within our biomarker signature has yet to be fully elucidated. Future studies combining carbon labelling of glucose with in vitro headspace analysis of primary cells will be required to more precisely establish the molecular origins of VOCs identified in this report.

Not all compounds were considered to be endogenous VOCs, with 27 possibly attributed to potential cosmetics. Eleven of the features predictive of the control group were assigned as either possible fragrances (e.g. alpha isomethyl ionone) or waxy long-chain chemicals used in cosmetics as emollients and surfactants (e.g. stearyl vinyl ether and isopropyl myristate). These may have been captured in the breath sample because of the proximity of the sorbent tubes to the patients’ faces. It should be noted that a frequent problem with ascribing the origin of VOCs is that those compounds often identified in cosmetics are natural products, therefore there is uncertainty about the precise origin of these makers. The downregulation in acute disease of several of these markers may be indicative of them being biomarkers as opposed to exogenous confounders from cosmetics.

Co-expression and enrichment analysis of the Louvain clusters on the correlation graph revealed a set of highly correlated metabolites significantly enriched in specific disease groups. Comparison of the Louvain clusters with the metabolite sets identified using the method previously described (7) demonstrated strong overlap. The metabolites enriched in heart failure were a cluster of highly correlated C_5-7_ hydrocarbons and C_3-5_ carbonyls with high chemical similarity (based on Tanimoto coefficients as determined in (fig. S10).The cluster included 2,4- and 2,2-dimethylpentane, 2-methylbutane, 2-methyl-1,3-butadiene (isoprene), 3-methylpentane, hexane and cyclohexane. These hydrocarbons (2,4- and 2,2-dimethylpentane, 2-methylbutane, and isoprene) have been individually reported and associated with heart failure and pneumonia (11, 14). However, the analysis herein captured the collective response and demonstrated enriched, co-expression of these hydrocarbons.

The analysis also revealed a separate set of highly correlated aldehydes (nonanal, decanal, undecanal, and a methyldecanal isomer), found to be potentially depleted in acute exacerbations of asthma compared with acute exacerbations of COPD and pneumonia. Depletion of VOCs during in vitro experiments has been reported as a consequence of metabolic activity by immune cells (18–20), but the association herein is tentative and should be interpreted with caution due to the correlation between inhaled air and exhaled air concentrations of these compounds (median Spearman rank = 0.60), also previously observed (21).

Our study has some limitations. Although internally replicated, the results presented here for acute VOC biomarker scores and cardiorespiratory exacerbation subtype biomarker scores are limited by the lack of external replication and internal replication respectively. The single centre design of this study may have introduced nonpathogenic biases related to diet, environment and lifestyle that might be absent in a multi-center study. The cardiorespiratory exacerbation disease subgroups pre-selected in this study were chosen as the commonest reported causes of cardiorespiratory breathlessness (22, 23) and there was a relatively high degree of clinical certainty in the diagnostic labels. For these findings to be generalisable, the identified markers will need to be validated in unselected cardiorespiratory populations and patients presenting with mixed acute pathologies.

In conclusion, we have conducted an acute care volatile breath biomarker study using robust clinical and analytical technology and have identified biomarkers with high combined diagnostic sensitivity and specify in acute cardiorespiratory disease. In addition, we have used methods enabling robust biomarker identification and mechanistic association. Future clinical studies in acute cardiorespiratory patients at initial presentation and triage using near patient sensor platforms capable of detecting the volatiles identified in this report are warranted to maximise the clinical impact of our discovery biomarker approach.

## 4 Materials and Methods

### 4.1 Study design

The study design, eligibility criteria and methodology have been described in detail previously (24). This is a prospective, real-world, observational study (ClinicalTrials.gov Identifier NCT03672994), carried out in a tertiary cardiorespiratory centre in Leicester, United Kingdom. Participants were recruited year-round from May 2017 through to December 2018.

Patients with self-reported acute breathlessness, requiring admission and/or a change in baseline treatment, presenting within University Hospitals of Leicester (UHL) were approached for study participation. Following triage and senior clinical assessment, if a primary clinical diagnosis of (i) acute decompensation of heart failure, (ii) exacerbation of asthma/COPD, or (iii) adult community acquired pneumonia was suspected by the triage nurse/attending clinician at triage, members of the research team would evaluate patients against predefined eligibility criteria for study participation.

A total of 277 participants were included in the final analysis. Sample size attrition from the recruited 455 participants is detailed in (Figure 1). This was mainly due to the delayed deployment of GCxGC-MS and analytical QC/QA. These decisions were made objectively during the discovery phase of the program, prioritising the optimisation of a robust sampling and analysis pathway. Sample size calculations were informed based on estimation for adequate sensitivity and or specificity as detailed in (table S1).

The 277 subjects were randomised *post-hoc* to Discovery and Replication cohorts in a 1:1 ratio through block random assignment. Randomisation was stratified based on (i) adjudicated clinical diagnosis, (ii) time to breath-testing from the point of hospital admission, and (iii) clinical diagnostic uncertainty score. The R package randomizr was used to perform block random assignment. After block randomisation there were 139 and 138 subjects in the discovery and replication sets respectively.

Inclusion and exclusion criteria and study objectives are outlined in detail in ‘study design’ and ‘study objectives’ sections of the Supplementary material. Informed consent was obtained in all participants within 24 hours of hospitalisation. Age- and/or home environment-matched healthy volunteers were recruited. Where environment-matched controls were unsuitable, healthy volunteers were recruited from local recruitment databases and via advertising. Healthy volunteers were defined as participants with no prior history of asthma, COPD, heart failure and had not been admitted to hospital with community acquired pneumonia within 6 weeks of the baseline study visit. The diagnostic accuracy of the reported exhaled breath VOCs was tested following the Standards for reporting of Diagnostic Accuracy Studies guidelines (25) (table S14).Statistical procedures presented here were carried out as complete case analysis with no imputations. Transparent Reporting of multivariate prediction model for Individual Prognosis or Diagnosis (TRIPOD) was followed for multivariate prediction models (28, 29) (table S15).

The trial was conducted in accordance with the ethics and principles of the deceleration of Helsinki and Good Clinical Practice Guidelines. All patients provided written consent. The National Research Ethics Service Committee East Midlands has approved the study protocol (REC number: 16/LO/1747). Integrated Research Approval System (IRAS) 198921.

### 4.2 Clinical adjudication

A clinical adjudication process was introduced to precisely define and quantify the diagnostic labels in the study, addressing any potential misclassification. A panel of two senior clinical adjudicators (SS & NG) reviewed all available case notes and imaging and determined the primary diagnosis for each case by discussion to reach a concordance. The degree of diagnostic uncertainty was marked on a 100-mm visual analogue scale (VAS scale), blinded to given diagnosis and blood biomarkers.

The process was implemented with emphasis on mirroring an acute triage pathway, where all pathology data required to support the diagnosis e.g. CRP, BNP are not available at the initial clinical review. The degree of diagnostic uncertainty obtained from the clinical adjudication process was factored into the block randomisation and subjects with higher diagnostic uncertainty (≥upper quartile = 20mm) were assessed separately as previously described (Figure 3C-D).

### 4.3 Breath collection and analysis

#### 4.3.1 Collection of breath samples

Exhaled breath collection was attempted in all consented participants using a CE marked breath sampling device ’Respiration Collector for In Vitro Analysis’ RECIVA (Owlstone Nanotech Ltd), in combination with a dedicated clean air supply unit (26). Breath sampling was well tolerated by all participants (6).

#### 4.3.2 Sample storage and preparation

Samples were dry purged on arrival for two minutes using nitrogen (chemically pure grade with inline trap, BOC) at a flow rate of 50 mL min^-1^ and then stored in refrigeration at 2 °C until analysis. Before analysis, samples were left to reach room temperature before being spiked with a 0.6 μL aliquot of 20 μg mL^-1^ standard solution containing deuterated toluene and octane, into a flow of nitrogen at a flow rate of 100 mL min^-1^ for 2 min, purging the excess solvent.

#### 4.3.3 Exhaled Breath analysis

Breath samples were analysed by thermal desorption with comprehensive two-dimensional gas chromatography (GCxGC) using flow modulation and coupled to dual flame ionisation detection and mass spectrometry (MS). Dual detection, with the use of MS and flame ionisation detection (FID), utilises the excess flow from the flow-based modulator suited for volatile analyses, providing both quantitative and qualitative results.

Analysis by GC×GC was optimised and conducted as described previously (5), using an Agilent 7890A gas chromatogram, fitted with a CFT flow modulator and 5799B mass spectrometer with a high efficiency EI ion source (Agilent Technologies Ltd). The instrument was coupled to a TD-100xr thermal desorption auto-sampler (Markes International Ltd). Samples were analysed in trays; typically six per tray along with a reference mixture containing n-alkanes and aromatics run every tray and a reference indoor air VOC mixture run every four trays. Data was acquired in MassHunter GC-MS Acquisition B.07.04.2260 (Agilent) and processed (i.e. baseline correction, alignment, feature extraction) with a workflow previously developed and optimised, using GC Image™ v2.8 suite (GC Image, LLC.) and Python (8). The sorbent tubes used were Tenax/TA with Carbograph 1TD (Hydrophobic, Markes International Ltd) with matching cold trap. Chromatographic features arising from analytical artefacts were removed from the peak table. (e.g. ubiquitous siloxanes). For purposes of quality control, samples were analysed in accordance with a previously published workflow and a detailed sample history, metadata and experimental data were recorded at every stage of the collection and analysis using the open-access LabPipe toolkit (5, 27).

#### 4.3.4 Chemical speciation of identified breath biomarkers

The chemical nature of volatile metabolites exhaled in breath comprises a diverse mixture of non-novel, low-molecular weight compounds. Thus, for most features, chemical identification involved comparison with an authentic reference compound in accordance with the Metabolomics Standard Initiative (MSI) Level 1 criteria for metabolite identification outlined in table S9. Identification was based on a minimum of two independent and orthogonal identifiers including primary and secondary retention time, mass spectral similarity match and calculated retention index. When an authentic reference compound was unavailable, chemical identification was compliant with MSI Level 2 for putative annotations. The highly structured chromatographic data and group-type separation afforded by GCxGC, alongside a well-characterised chromatographic space from analysing an extensive library of authentic compounds, gave increased confidence in the tentative assignments made. The orthogonal separation of GCxGC also meant chemical identification of unknown metabolites could be made, at minimum, in compliance with MSI Level 3 for putative chemical classification.

#### 4.3.5 Sample analysis quality control/quality assurance (QC/QA) procedures

For purposes of quality control, samples were analysed in accordance with a previously published workflow and a detailed sample history, metadata and experimental data were recorded at every stage of the collection and analysis using the open-access LabPipe toolkit (27). The chromatographic method was optimised for peak shape, sensitivity and separation; quality control charts of the internal standards were used to track the stability of the TD-GCxGC-FID/MS analysis, and instrument performance was evaluated following the assessment of the variation of retention times, peak area and shapes of VOCs in two standard reference mixtures every six samples (5). Before being conditioned and sent to clinic, the number of heat cycles and weight for each tube was recorded to monitor tube age and integrity. For each conditioning cycle, all tubes were given a batch number and a batch blank was analysed to monitor contamination from the beginning of the sample preparation process. Furthermore, all batches were given an expiry of two weeks to ensure routine monitoring.

To minimise the influence of biological and analytical confounders (e.g. circadian rhythm, sample stability), potential effects due to the operator, date of analysis, time of day collected, storage time before dry purging, sample storage time after dry purging and collection volume were assessed and where necessary accounted for in the batch correction. In addition to the routine analysis of reference standards, used to monitor retention shift and instrument response, the TD-GCxGC analytical system underwent a programmed heat cycle between each sample to reduce potential issues arising from sample carry-over, and a TD-trap blank and empty sorbent tube were analysed every six samples to monitor the instrument baseline signal.

#### 4.3.1 Topological data analysis in the discovery and replication sets

In topological data analysis, the x-y coordinate position of a particular patient within a TDA cluster cannot be directly compared between discovery and replication TDA graphs, as the graphs represent a simple 2-dimensional projection of a higher dimensional structure. Prior to performing TDA, each feature was *log*(*x* + 1) transformed. TDA parameters were set as: number of hypercubes=20, where the number of hypercubes refers to the number of overlapping intervals of the projection.

The distance between data points was measured using the Euclidean distance. The first two linear discriminant functions (LD1) and (LD2) were used as the projection. Clustering on the overlapping intervals on the projection was done using agglomerative (bottom up) hierarchical clustering with complete linkage. TDA was performed using Kepler Mapper 1.4.0 (36) with Python 3.5.

Herein, we computed the equivalence between topological data shapes generated using 805 volatile features extracted from the GCxGC-MS peak data, in both the discovery and replication cohorts.

#### 4.3.2 Breath biomarker score generation

Feature selection was implemented via Lasso and Elastic-Net Regularized Generalized Linear Models (GLMNET) using the glmnet package in R. After removing features present in <80% of all samples from the (*x* + 1) transformed discovery GCxGC-MS peak data a 735-feature matrix was obtained. A multinomial regression model using LASSO regularization was fitted to the 735-feature matrix in the discovery set using 10-fold cross validation, with the dependent variable in the model being clinical diagnosis (acute asthma, acute COPD, pneumonia, heart failure, or healthy volunteers). The 10-fold cross validation was repeated 100 times; features that had a non-zero regression coefficient in more than 80 of the cross validation runs were considered as being stable candidate features predictive of the outcome (clinical diagnosis), and this resulted in 278 stable candidate features. For validation, predictors were calculated using the *Predict Function* of (GLMNET).

A multinomial regression model using elastic net regularization was fitted to the 278 features with the dependent variable in the model being clinical diagnosis. Following the chemometric inspection detailed above and the lasso and elastic regression analysis, a final set of 101 exhaled breath volatile compounds was generated.

A multinomial regression model using elastic net regularization was fitted to the matrix of 101 breath biomarkers with the 10-fold cross validation repeated 100 times. The R package glmnetUtils was used to determine the optimal value of αthe elastic net penalty, the best value for α was 0 (Ridge regression). Ridge regression with a logit link function (binary logistic regression) was fitted to the 101 breath relevant features; the dependent variable was ‘acute disease’, as a binary outcome. The linear predictor from the combination of the most stable features was used to as a score to predict acute disease. Linear combinations of the most stable features from the multinomial regression model fitted to the 101 biomarkers formed a set of scores for predicting probability of belonging to the different disease groups (acute Asthma, acute COPD, pneumonia, heart failure or healthy volunteers). Sensitivity analysis for the interactive elastic net regression approach and justification of the optimal α values are provided in (figs. S11-S12 and tables S6-S8).

Figure S13 is a graphical probability distribution of the final 101 exhaled breath features in the GCxGC-MS peak data. The features largely follow a similar distribution. Some features contained a mixture of zero and non-zero values, which have arisen owing to the measurement being below the instrument’s lower limit of detection. Constant features (all zero values) were removed prior to fitting the main model.

#### 4.3.3 Breath biomarker co-expression and feature enrichment analysis

It was of interest to investigate if within the final set of 101 features, sets of ‘co expressed’ features existed, i.e. sets containing features that are correlated. Considering sets of co-expressed features has value in terms of reducing the dimensions of a problem and mitigating the multiple testing problem through the use of enrichment score. Co-expression and feature enrichment analysis are described in the (Supplementary material section ‘co-expression and feature enrichment analysis’). Metabolite sets were derived based on Ward hierarchical cluster analysis using the ChemRICH method reported by (7), and broader communities were derived from Louvain cluster analysis to help interpret the correlation graphs (Supplementary material section ‘co-expression and feature enrichment analysis’). Covariation among metabolites lacks evidential value on its own, therefore, set-level significance was established using the Kolmogorov-Smirnov test (K-S test) as described using the ChemRICH method (7), Tanimoto coefficients were calculated to asses intra-set chemical similarity using Metabox (37), and the frequency of occurrence in the published literature and relevant databases considered (KEGG, ChEBI, Human Metabolome Database, Human Breathomics Database and microbial VOC database). Chemical similarity is of interest because compounds derived from similar pathways may also share common structural features or chemical groups. This combined data-driven and chemistry-driven approach has been shown to improve enrichment analysis (7, 38), and allowed further interpretation of core findings herein (fig. S10).

### Statistical procedures

Statistical analysis was performed using R [3.6.1 and 4.0.0, R Core Team (2019)]. This research used the SPECTRE High Performance Computing Facility at the University of Leicester. Baseline data and figures were presented as mean ± (SD), and median (IQ range). Data was analysed using (ANOVA) to assess the differences between groups for normally or approximately normally-distributed variables and Kruskal-Wallis for non-normally distributed variables. Pearson chi-squared and Fisher’s exact were used to assess the differences in categorical variables. All *P* values are two sided and significant at the 0.05 level, unless reported otherwise.

## Supplementary Material

Supplementary aux table 1

Supplementary material

## Figures and Tables

**Table 1:** Demographics and clinical characteristics of study participants. **C**ontinuous variables are presented as mean ± standard deviation. Categorical variables are presented as numbers (%).

	Total number	Healthy controls	Acute asthma	Acute COPD	Pneumonia	Heart failure	*p* value
**Total number of participants (n=**)	**277**	**55**	**65**	**58**	**55**	**44**	
**Demographics**							
Age *, years	60.8 ± (16.8)	63.05 ± (11.78)	44.3 ± (17.93)	69.82 ± (8.16)	60.67 ± (16.50)	70.72 ± (11.04)	.124
Gender Male (n=) (%)	143 (51%)	26 (47%)	25 (38%)	33 (56%)	27 (49%)	32 (72%)	**.008 ¥**
Body Mass Index (BMI)*^a^	29.5 ± (7.3)	28.2 ± (4.5)	31.5 ± (9.0)	27.5 ± (7.7)	29.2 ± (6.9)	31.5 ± (6.5)	.767
Smoking Current smoker (n=) (%)	53 (19%)	4 (7%)	13 (20%)	21 (36%)	11 (20%)	4 (9%)	**.001 ¥**
**Vital signs**							
Temperature (Celsius)*	36.7 ± (0.6)	36.1 ± (0.4)	36.8 ± (0.5)	36.7 ± (0.5)	37.1 ± (0.7)	36.5 ± (0.3)	**.000**
Heart rate (beats/min)*	87.2 ± (18.5)	68.1 ± (9.54)	99.6 ± (17.2)	92.9 ± (15.6)	90.3 ± (15.4)	81.3 ± (15.6)	**.005**
Respiratory rate (breaths/min)*	18.9 ± (4.2)	13.0 ± (1.8)	20.5 ± (3.4)	21 ± (2.5)	20.4 ± (4.6)	19.1 ± (1.8)	**.000**
Oxygen saturations (%)*	95.8 ± (3.0)	97.7 ± (1.3)	96.1 ± (2.5)	94.0 ± (2.9)	94.5 ± (0.5)	96.5 ± (1.9)	**.001**
Systolic Blood Pressure (mmHg)*	131.5 ± (19.2)	134 ± (15.7)	133 ± (17.7)	133 ± (20.5)	126 ± (19.4)	128 ± (22.2)	.515
Total mEWS-2 score ^^b^	1 (0-3)	0 (0-1)	2 (1-3.5)	3 (1-5)	2 (1-3)	1 (0-2)	**.000**
**Breath sampling**							
**Time from admission to breath sampling (hours)^**	16 (3.0−23.0)	1 (1-1)	16 (9.2−22.7)	18 (12.5-23.0)	18 (11.0-23.0)	23 (19.0-26.0)	.000
**Symptoms assessment**							
Breathlessness VAS score (mm)*^c^	58.1 ± (31.6)	6.2 ± (9.3)	76.6 ± (14.2)	71.6 ± (19.2)	67.8 ± (22.1)	67.9 ± (20.0)	.000**
Cough VAS score (mm) *^c^	43.3 ± (33.2)	8.7 ± (14.3)	64.5 ± (26.7)	57.8 ± (27.0)	53.6 ± (30.6)	24.3 ± (25.2)	.000**
Wheeze VAS score (mm) *^c^	41.8 ± (34.9)	3.4 ± (6.4)	66.2 ± (24.5)	60.3 ± (29.0)	45.1 ± (34.8)	28.1 ± (28.6)	.000**
**eMRCd score (n=) (%)**							
**1**	17 (6%)		1 (1.5%)	8 (13%)	7 (12%)	1 (2%)	.000¥
**2**	6 (2%)		0 (0%)	0 (0%)	5 (9%)	1 (2%)	.000¥
**3**	15 (5%)		6 (10%)	0 (0%)	7 (12%)	2 (4.5%)	.000¥
**4**	50 (18%)		16 (25%)	11 (19%)	6 (11%)	17 (38.5%)	.000¥
**5a**	112 (40%)		38 (51%)	32 (55%)	22 (41%)	20 (46%)	.000¥
**5b**	21 (7%)		3 (4.5%)	7 (13%)	8 (15%)	3 (7%)	.000¥
**Exposure to antibiotics and steroids within 2 weeks of hospital admission**							
**Antibiotics (n=) (%)**	61	n=0 (0%)	n=24 (36.9%)	n=23 (39.6%)	n=10 (18.2%)	n=4 (9.0%)	.002¥
**Steroids (n=) (%)**	57	n=0 (0%)	n=28 (43.0%)	n=24 (41.3%)	n=3 (5.4%)	n=2 (4.5%)	.000¥
**Morbidity and mortality measures**							
**Length of hospital stay (days) ^**	3 (2-6)		2.0 (1.0-3.0)	4.0 (2.0-6.0)	4.0 (2.0-5.0)	7.0 (4.0-11)	.000**
**30-60 days hospital readmission (n=)**	29		7	9	6	7	.461¥
**1-year all-cause mortality**	12	0	1	5	1	5	.078¥
**Laboratory parameters**							
**C-reactive protein (CRP) (mg/L)^**	11 (5.0-34.2)	5 (5-5)	10.0 (5.0-23.0)	12.0 (5.0-20.7)	108.0 (53.5-245.3)	11.0 (5.0-22.0)	.000**
**Blood Eosinophil count 109/L^**	0.13 (0.06-0.24)	0.17 (0.09-0.24)	0.18 (0.06-0.42)	0.13 (0.06-0.24)	0.08 (0.04-0.14)	0.13 (0.08-0.23)	.000**
**Troponin T (ng/l)^**	3.3 (1.0-11.4)	2.05 (1.0-2.7)	1.55 (1.0-3.4)	3.75 (2.6-10.9)	4.3 (2.18-11.3)	20.2 (13.4-59.6)	.000**
**Brain natriuretic peptide (BNP) (ng/l)^**	40.5 (20.6-98.9)	28.40 (17.60-39.88)	20.4 (12.1-40.0)	56.3 (24.3-95.0)	56.3 (27.4-132.1)	611.8 (172.1-1259.1)	.000**
**Questionnaires**							
Asthma Quality of Life Questionnaire (AQLQ) total*	65		117.3 ± (37.3)				
COPD Assessment test (CAT) *	58			26.7 ± (7.3)			
COPD Decaf score *	58			1.7 ± (0.8)			
**CURB65 score^**	55				2 (1-3)		
**NYHA score^**	44					2 (1-3)	

a The body mass index (BMI) is the weight in kilograms divided by the square of the height in meters.

b Modified Early warning score - 2 (MEWS-2) is a guide widely used by medical services to determine the degree of illness of a patient based on their vital signs including respiratory rate, oxygen saturations, temperature, blood pressure, and heart rate. Vital signs collected at the point of admission for acute disease groups.

c Participants were asked to determine their degree of breathlessness, cough and wheeze on a 100mm visual analogue scale (VAS) on admission. Higher scores indicate worse symptoms.

d Extended Medical research Council (eMRC) scale is a validated measure of perceived respiratory disability, scored from 1 to 5b. Higher scores indicate worse disability.

* Data is expressed as mean (SD) or n (%) ± (SD)

^ Data expressed as median (IQ range)

** Kruskal-Wallis test comparing non-parametric data, ¥ Pearson Chi Squared and Fisher’s Exact test.

ANOVA was used to assess the differences between groups for normally distributed continuous variables and Kruskal-Wallis for non-parametric continuous variables. Pearson chi-squared and Fisher’s exact were used to assess the differences in categorical variables. The results were considered statistically significant at *p*-values <0.05.

## Data Availability

All data associated with this study are available in the main text or the supplementary materials. R and Python codes have been archived in Zenodo (https://doi.org/10.5281/zenodo.6956451).

## One sentence summary

Exhaled VOCs can distinguish acute cardiorespiratory exacerbations and associated subtypes and map underlying metabolic clusters.
