# Supplementary material for: Visualisation of exhaled breath metabolites reveals distinct diagnostic signatures for acute cardiorespiratory breathlessness
Source: Sci Transl Med. Author manuscript; Available in PMC 2022 Nov 28. (PMC7613858; doi:10.1126/scitranslmed.abl5849)
Supplement: Supplementary material [file EMS156250-supplement-Supplementary_material.docx]

**Supplementary material for**

**Visualisation of exhaled breath metabolites reveals distinct diagnostic signatures for acute cardiorespiratory breathlessness**

**†Corresponding authors:**

**Dr Michael J Wilde**

Lecturer in Analytical & Environmental Chemistry

School of Geography, Earth and Environmental Sciences

Faculty of Science and Engineering

University of Plymouth

E-mail: michael.wilde@plymouth.ac.uk

t (direct): +44 (0)1752 584793

**Professor Salman Siddiqui BM, FRCP, PHD**

Professor of Respiratory Medicine

National Heart and Lung Institute

Imperial College,

St Mary’s Campus

Norfolk Place

London, W2 1PG

E-mail: [s.siddiqui@imperial.ac.uk](mailto:s.siddiqui@imperial.ac.uk)

**This file includes:**

Materials and Methods

Supplementary Figures S1 to S13

Supplementary Tables S1 to S15

# Materials and methods

## Study design

This clinical study was a prospective, real-world, observational study, carried out in a tertiary cardiorespiratory centre in Leicester, United Kingdom. Participants were recruited year-round from May 2017 through December 2018.

Recruitment started in February 2017 and, following analytical method development and optimisation of a robust sample pathway for achieving continual deployment, collection and analysis of sorbent tubes to-and-from clinic, the analysis of samples by GCxGC-MS was set up and brought online later that year in August 2017. The denominator for the entire study was 455 participants and for the GCxGC-MS study presented here was 363 participants, with a 76% GCxGC-MS completion rate.

**(Fig. S1)** is a visual abstract representing the proposed breath testing and diagnostic pipeline.

Traceable and verifiable quality control and quality assurance (QC/QA) procedures have been applied throughout the breath sampling and analysis steps. These are detailed in section 4. The number of breath samples fulfilling all QC/QA criteria is outlined in **(fig. S2).**

Inclusion criteria

1. Able to give informed consent for participation in the study.
2. Male or Female, aged 16 years or above (adult cohort) and 5-15 years for paediatric patients attending the acute care paediatric pathway.
3. Capable [in the opinion of the study clinical research investigator(s)] of providing serial breath samples.
4. Diagnosed with acute breathlessness as one of the primary indicator reasons by the clinical acute care team. This is not a requirement for healthy subjects or matched controls.
5. One of the indicator provisional diagnoses (acute asthma exacerbation, acute COPD exacerbation, heart failure exacerbation, and community acquired pneumonia) following senior review by the clinical acute care team. This is not a requirement for healthy subjects or matched controls
6. Able (in the Investigators’ opinion) and willing to comply with all study requirements.
7. Willing to allow his or her General Practitioner and consultant, if appropriate, to be notified of participation in the study.
8. Ability to understand English.

Exclusion criteria

1. Female participants who are known to be pregnant, lactating or planning pregnancy during the course of the study.
2. Current participation in a clinical trial of an investigative medicinal product (IMP) or within 3 months or 5.5 half-lives of the IMP whichever is longer.
3. Active or clinically suspected pulmonary tuberculosis
4. In the opinion of the treating physician, breath sampling during the acute admission would be clinically unsafe or inappropriate due to the patient’s condition or poor prognosis. Examples include malignancy or autoimmune disease with anticipated survival of under 1 year, and chronic renal replacement therapy.
5. Unable or unwilling to give informed consent
6. Any other significant disease or disorder which, in the opinion of the Investigator, may either put the participants at risk because of participation in the study, or may influence the result of the study, or the participant’s ability to participate in the study.

## Study objectives

The primary and secondary objective of the EMBER study are summarised below.

Primary objective

- To evaluate the sensitivity, specificity, positive and negative predictive values of exhaled breath VOC biomarkers to differentiate acute breathlessness in cardiorespiratory patients.

Secondary objectives

- To replicate selected breath VOC biomarkers identified in acute breathlessness.
- To discover and replicate breath VOC biomarkers that differentiate the common cardiorespiratory conditions that cause acute breathlessness, specifically: (1) acute heart failure, (2) community-acquired pneumonia, (3) adult exacerbations of asthma and chronic obstructive pulmonary disease (COPD) and age-matched adults that do not have cardiorespiratory disease or breathlessness.
- To quantify the level of clinical uncertainty in the primary diagnosis using a 100 mm visual analogue scale (VAS) and independent clinical adjudication of case notes blinded to the following blood biomarkers: (1) CRP, (2) BNP, (3) troponin-I and (4) blood eosinophils but not clinical history and acute presentation nor chest X-ray imaging. Potential discriminatory breath VOC biomarkers will be adjusted for clinical uncertainly in statistical models.

## Statistical analysis

### Sample size estimation

Based on a total sample size of n=277 *post-hoc* sample size calculations were performed. We assumed an 80% acute disease prevalence for recruitment and 1:5 patients recruited were non- breathless healthy controls. We were powered to identify a breath biomarker VOC score of acute cardio respiratory exacerbations demonstrating combined diagnostic accuracy (sensitivity and specificity) of ≥ 80%, with a precision of 95% and maximal marginal error of ±15% in discovery and replication cohorts **(table S1)**.

Data collection was stopped when a minimum of 55 participants were recruited to each disease group. Details on participants’ clinical characteristics and comorbidities are outlined in **(tables S2-3).**

### Sensitivity analyses - Impact of zero valued peak areas and removal of features present in < 80% of participants on classification accuracy

We ruled out features not present in >80% of samples to effectively reduce the number of features used in subsequent models with more than 20% of 0s (imputed values). In extreme cases where the feature contained nearly all 0s there is no useful information, the feature is essentially constant and can reasonably be excluded. We found that the 0s were randomly distributed across the disease groups in all but 7 features. The exclusion of the 7 features where there was some evidence that 0s were not randomly distributed across the disease groups did not alter the results of the regression models.

We fit the LASSO to the full feature matrix (805 x features), then fit the LASSO to the feature matrix with the 7 features **(table S4).** There appeared to be no substantial change in the performance of the model after excluding the 7 features. Removal of the 7 features did not result in a substantial improvement in model performance.

We considered the effect on model performance after removing all features present in less than 80% of samples **(table S5).** By removing all features present in less than 80% of samples (the approach we have adopted in this manuscript) we saw an improvement in model performance. For example, the miss- classification error decreased from approximately 60% to approximately 19%.

**Tables S4-S5** were produced using output from assess.glmnet from the glmnet package in R.

### Elastic net regression sensitivity analyses

The solution to the elastic net is (𝛽0̂,̂), where:

(𝛽0̂,̂)=(𝛽0̂,𝛽̂)∈ℝ𝑝+1[12𝑛Σ(𝑦𝑖−𝛽0−𝑥𝑖𝑇𝛽)2𝑛𝑖=1+𝜆(1−𝛼2‖𝛽‖22+𝛼‖𝛽‖1)]

The hyper-parameters (λ) and (α) control the overall penalty and the elastic net penalty respectively. We do not as a rule chose a value for λ; instead a default set of values (0 to 100) is used for λ. We can choose a value for α in the range 0 to 1. If we set α=0, then we have Ridge regression (the elastic net becomes Ridge regression), if we set α=1, then we have the LASSO (the elastic net becomes the LASSO). Setting α to close to 1 (but not equal to 1), will provide a sparse solution, setting alpha close to 0 (but not equal to 0) will provide a far less sparse solution. By sparse we mean the number of variables with regression coefficients that are shrunk to 0. Note for α=0 we cannot achieve a sparse solution, the regression coefficients are shrunk but will never be set to 0.

## Breath collection and analysis

### Breath collection

Breath collection was carried out using a CE marked breath sampling device ’Respiration Collector for *In Vitro* Analysis’ RECIVA (Owlstone Nanotech Ltd), which aims to standardise the collection of alveolar breath by providing the patient with a VOC-clean air supply; controlling the flow, volume and fraction of breath collected, while directly sampling the exhaled VOCs onto the sorbent tubes. The ReCIVA settings mode was set to ‘lower airways only’, the continuous monitoring of the CO2 and partial pressure allowed targeting the VOC-enriched alveolar fraction of breath. The collection volume, flow rate and maximum sampling time were set to 1 L, 250 mL min^-1^, and 900 seconds respectively.

At the time of sampling, the room air and air supply were also sampled as environmental controls. This involved attaching a sorbent tube to a handheld personal pump (Escort Elf, Sigma Aldrich, Dorset, UK) and having the sampling end either open to the room air or attached to the ReCIVA air supply line via a T-piece. 1 L of air was collected in total at a flow rate of 0.5 L min^-1^ for 2 min. Sorbent tubes were immediately capped (brass caps, Markes International Ltd) and placed in a refrigerator at 4 °C before being dispatched to the laboratory within 72h.

To minimise background variation, sample collection was completed, when possible, in the same treatment room attached to the admissions ward. Unwell patients and those requiring supplemental oxygen, however, had their samples collected by their bedside.

### Analysis of room air and air supply samples

Two separate elastic net regression models were fitted to peak tables for room air and air supply samples, both peak tables where 𝑙𝑜𝑔𝑒(𝑥 + 1) transformed and adjusted for batch effects (collection date) using Parametric Empirical Bayes Adjustment (PEBA). The independent variables were the final set of 101 features and the dependent variable was clinical diagnosis (acute asthma, acute COPD, pneumonia, heart failure or healthy volunteers). After repeating 10-fold cross validation 100 times for each of the two models, only two features were found to have stable non-zero regression coefficients. These features were for air supply, a component of the pneumonia score and for room air, a component of the healthy score, highlighting the robustness of the selected feature separation models.

### Mitigating the adverse impact of batch effects in biomarker pattern detection

Batch effect is a common issue in omics data analysis. The existence of batch effects makes it challenging to compare data collected and analysed at different processing times. **(Figures S3-4).**

We sought to investigate the following factors as possible contributing batch variation factors:

**I. Batch_ID - date of sample collection:**

**(1)** Batch 1 – August 2017 - October 2017

**(2)** Batch 2 – November 2017 - March 2018

**(3)** Batch 3 – April 2018 – December 2018

**II. Operator:** (N: 1-6) – indicating members of the study team operating the RECIVA over the entire course of the sampling program

**III. Time of the day sample was collected (circadian rhythm):**

(1) 1 = between 9-11am

(2) 2 = between 11am-1pm

(3) 3 = between 1-3pm

(4) 4 = between 3-5pm

**IV. Time sample stored wet**

(1) 1 = 0-2 days

(2) 2 = 2-5 days

(3) 3 = 5-10 days

(4) 4 = 10-20 days

(5) 5 = 20-42 days

(6) 6 = over 42 days

**V. Time stored dry (following dry purging)**

(1) 1 = 0-2 days

(2) 2 = 2-5 days

(3) 3 = 5-10 days

(4) 4 = 10-20 days

(5) 5 = 20-42 days

(6) 6 = over 42 days

Wet storage refers to the time sorbent tubes were stored (at 4°C) prior to dry purging (typically <48 hours) and dry storage refers to the time the sorbent tubes were stored (at 4°C) after dry purging (up to 6 weeks).

Samples were dry purged to remove excessive moisture, which condenses in the sorbent tube during sample collection. The presence of excessive moisture during thermal desorption can cause inaccuracies in sample injection due to the rapid expansion of vapour, disrupting the carefully controlled gas flows. Dry purging the sorbent tubes removes the condensed moisture, up to 5 mg in weight. Every endeavour was made to dry purge samples within 48 hours of collection to reduce potential detrimental effects of the initial high water load on the sorbent material and adsorbed VOCs such as hydrolysis.

**VI. Volume of breath collected (over 80% threshold):**

(1) 1 = 100% 1357

(2) 2 = 90-99% 1358

(3) 3 = 80-89%

The % refers to the % of acquired breath using the ReCIVA sampler onto the TD tube of the target breath volume of 1 Litre. This was a pragmatic real world acute study in acutely ill patients (who sometimes could not be sampled for long enough to acquire ≥ 0.8 L of breath). To reduce the risk of reduced data quality we excluded patient sample that were less than the target collection of 0.8 L (see consort diagram Fig S1, *n=28*). The selection of ≥ 0.8 L as a minimum threshold ensured sufficient pre-concentration of exhaled compounds. Because the collection of breath VOCs on sorbent tubes is a pre-concentration technique, use of a threshold avoids artificial skewing as a result of scaling breath profiles with insufficient volume. The threshold used was consistent with the recommended threshold of the manufacturer, Owlstone Medical.

**Figure S3** is a visualization of the GCxGC-MS peak table comprising all 805 features using t Stochastic Nearest Neighbour Embedding (tSNE) [5]. Clustering due to ‘date of collection’ was seen (top left plot). No obvious clustering seemed to be present for the remaining factors. The effect collection date was adjusted for by applying Parametric Empirical Bayesian Adjustment (PEBA). The ComBat function from the SVA package for Bioconductor was used to perform PEBA. The results of this adjustment are shown in **figure S4.** It can be seen that the clustering due to collection date is no longer apparent. The batch effect adjusted peak table was used in all subsequent feature selection models.

### Quality control and quality assurance systems

Traceable and verifiable quality control and quality assurance (QC/QA) procedures have been applied throughout the breath sampling and analysis steps. This ensured efficient prevention of any anticipated defects and high deliverable standards.

To eliminate any samples from the final analysis that were of poor quality four criteria were used to selected for high quality breath samples. These were:

1) ≥800 mL of breath collected from the patient to ensure sufficient pre-concentration of trace

VOCs present in breath.

2) The concentration of isoprene and acetone in the air supply were ≤ 3 standard deviations of the mean air supply concentration. This ensured that no breath samples were mis-assigned as air supply samples.

3) The concentration of isoprene and acetone in breath were ≥10 and ≥ 5 standard deviations, respectively, above the concentrations measured in the patient air supply. This ensured that the samples were not mis-assigned air supply samples, and that breath had been collected onto the sorbent tubes

4) The chromatogram, on visual review, was not distorted by an abundance of exogenous compounds (i.e. overloaded peaks).

### Chemical speciation of identified breath biomarkers

To confirm the chemical identity of the concatenated list of 101 exhaled breath peaks, standard reference compounds, where available, were purchased and analysed**.** This included a C8- C20 saturated alkanes certified reference material (Sigma Aldrich, Dorset, UK), an aromatics calibration standard (NJDEP EPH 10/08 Rev.2, Thames Restek, Saunderton, UK), a multi-component indoor air standard (Sigma Aldrich, Dorset, UK), two terpene reference mixtures (Spex Centriprep, Emerald Scientific, San Luis Obispo, US), and individual standards from Sigma Aldrich (Merck Life Sciences), Greyhound Chromatography, Scientific Lab Supplies, Alfa Chemicals and Santa Cruz Biotechnology.

Table S9 lists the chemical assignment of the selected predictive markers from the regression model detailing chemical name, CAS registry number, KEGG, Human Metabolome Database and ChEBI identifiers and MSI-compliant metabolite identification level, concentration range and fold change (expressed as log2) between acute and control groups, and compound contribution towards disease-specific biomarker risk scores (†adjusted p-value <0.05).

### Breath biomarker score classification accuracy

The overall classification accuracy for the statistical model generated from 101 breath biomarkers was assessed by comparing the balanced accuracy of model trained using the true class labels versus the balanced accuracy of the same model tested using randomly shuffled class labels. This process was repeated 1,000 times. The balanced accuracy is reported in **(Fig. S6)** for (A) the acute disease biomarker score in the discovery cohort, (B) the acute disease biomarker score in the replication cohort and (C) the multinomial biomarker scores for the five subgroups acute asthma, acute COPD, heart failure, pneumonia and healthy volunteers. NB: replication was not evaluated in the subgroups as the study was not powered to do this.

Receiver operating curve (ROC) analysis to assess the diagnostic value of the heart failure biomarker score against other acute disease groups was AUC: 0.78 (0.70-0.86) *P* <0.0001, sensitivity 0.77 (0.64-0.89), specificity 0.71 (0.64-0.78), PPV 0.40 (0.29-0.50), NPV 0.92 (0.88-0.97) (**fig. S7).**

The median values of the exhaled breath VOC scores and their distribution across disease subgroups are detailed in **(fig. S8). Figure S9** is a Venn diagram demonstrating the distribution of the final panel of 101 exhaled breath biomarkers across the different disease groups.

### Co-expression and feature enrichment analysis

**Graph construction and Louvain cluster analysis**

Subjects from both the Discovery and Replication sets were combined into a data matrix M_D_ comprising the 101 features that were obtained from previous regression analysis, with healthy 1483 subjects excluded. The Spearman rank correlation matrix was calculated for the data matrix MD.

A scale free graph g was constructed by generating the adjacency matrixMAdj=|Ĉ|β.

Where Ĉ is the sample correlation matrix ofMD, and β≥1.

The pickSoftThreshold function from the WGCNA package in R was used to estimateβ. The igraph 1487 package in R was used to construct g usingMAdj, g is a weighted and unsigned graph. The graph g 1488 will be referred to as the “correlation graph”.

Louvain clustering was then performed on the correlation graph and 8 feature sets were obtained **(fig. S10).**

The 8 feature sets obtained from Louvain clustering on correlation graph were used in an enrichment analysis. Instead of considering individual features and how they might distinguish different disease groups, sets of features are considered, the idea being that features in combination may have better discriminatory capability. The Bioconductor (version 3.12) packages GSVA and limma were used to perform enrichment analysis. Feature set 3 was found to be enriched in Asthma and HF, feature set 5 was found to be enriched in HF alone, see **(tables S10-S13).** The enriched feature sets 3 and 5 did not demonstrate improved diagnostic accuracy over the scores obtained from regression analysis **(fig. S10).**

### The approach taken to modelling the multinomial response was as follows:

1) Remove features that were present in less than 80% of the total samples.

2) Fit the elastic net model (α =1, LASSO) to the remaining features, with a view to candidate biomarker variable reduction.

3) Obtain the active set (features with non-zero coefficients) from step 2.

4) Fit an elastic net model to the active set from step 2 with α determined by performing 10-fold cross-validation on the active set from step 2; here α was found to be 0.5 approximately, simulated range from 0.6-0.7 **(fig. S11).**

5) Obtain the active set from step 4, fit an elastic net model with α determined by performing 10-fold cross-validation on the active set from step 4; here α was found to be 0 approximately, simulated range 0-0.15 **(fig. S12).**

We then examined the model performance as we iterated the regressions across iterations 1-3

From **tables S6-S8**, we see that the misclassification error decreases from approximately 20% at iteration 1 to approximately 7% at iteration 3. The logic behind iterations 1-3 is to fit the LASSO initially to remove the gross noise and reduce the dimensions of the problem. Once the noise has been removed the remaining features may be more likely to contain useful information, so we fit an elastic net model with a less harsh penalization, but still we still try to reduce the number of features. The last iteration fitting the Ridge model performs shrinkage but does not set coefficients to 0. There may be situations where LASSO followed by Ordinary Least Square (OLS) is not possible, i.e. features in the active set from the LASSO are correlated (multi-collinearity). In this case, LASSO followed by Ridge regression would be a valid approach.

In applying the LASSO initially and then again to the active set a single hyper-parameter λ is estimated by cross-validation (for the initial LASSO and then for the subsequent LASSO on the active set) using the default grid 0-100 for λ. To estimate the optimal α using glmnetutils or ensr sequential application of a penalized model is performed.

### Probability distributions of breath features

The features in the GCxGC peak table fell into 3 broad categories: (1) constant features (all samples had a value of zero), (2) features that contained a mixture of zero and non-zero values, and (3) features that contained all non-zero values. The zero values arose because the measurement was below the instrument’s lower limit of detection. Constant features were removed prior to fitting the main model.

Graphical distribution of the final 101 features, mainly falling into type 2 and 3 categories, is illustrated in **(fig. S13).** For certain features the spike in the 0 values can be clearly seen. Based on these observations a reasonable choice for a theoretical model for the probability distribution of a feature from a GCxGC-MS peak table might be the Zero Modified Log Normal distribution.

# Supplementary Figures and legends:


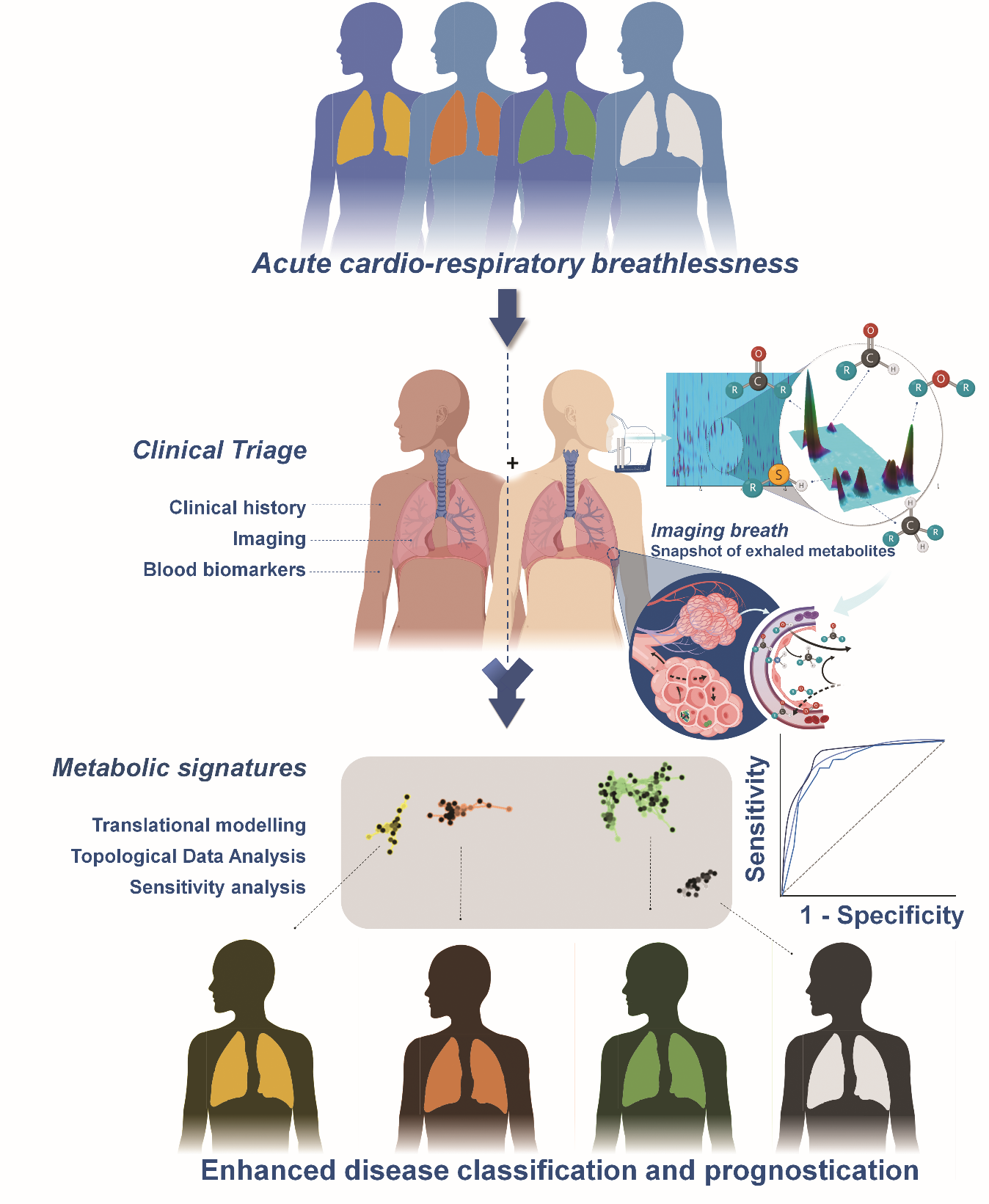


**Figure S1.** **A visual abstract representing the proposed breath testing and diagnostic pipeline.**

Acutely breathless patients with cardiorespiratory disease exacerbations are currently triaged on admission by means of clinical assessment, digital pathology, and blood biomarkers. Lower airway-derived breath volatile organic compound (VOC) biomarkers, visualised using state of the art GCxGC mass spectrometry, undergo a process of chemometric and translational modelling. The resultant breath metabolic signatures provide accurate disease classification in acute cardiorespiratory patients, with co-location of specific VOC profiles and VOC classes with individual exacerbation subgroups. Breath biomarker score generated using Elastic Net Regression were used to evaluate the diagnostic accuracy of breath VOCs in acute cardiorespiratory disease. Created in part with BioRender.com.

**
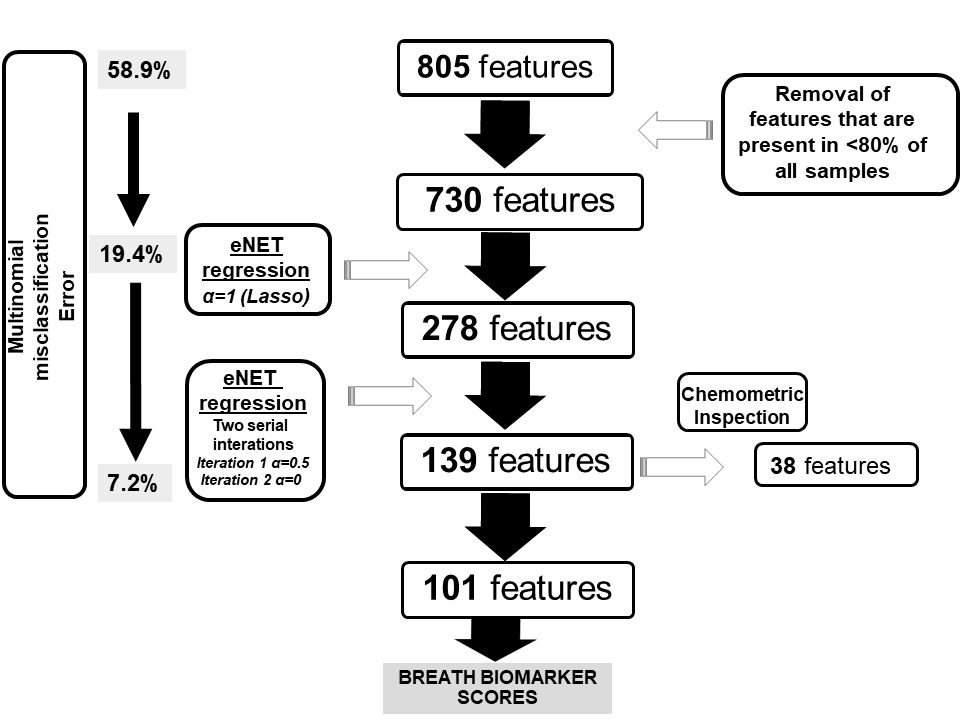
**

**Figure S2. Exhaled breath features flow chart.** Flow chart demonstrating the removal of exhaled breath features from 805 to 101 breath GCxGC biomarkers for risk score generation. An iterative elastic net (eNET) modelling approach (across three iterations) was adopted as the feature selection methods of choice owing to the high variables to subject ratio and the potential correlations among the candidate features. The attrition of the misclassification errors is demonstrated in the left hand panel.





**Figure S3. A 2-dimensional visualization of the high dimensional peak table before adjustment for batch effects.** Clustering by date of collection ‘Batch_ID’ in the first panel can be clearly seen, compared to other variables (operators, time of collection, time of wet and dry storage, and collection volume) where no batch effects are apparent.


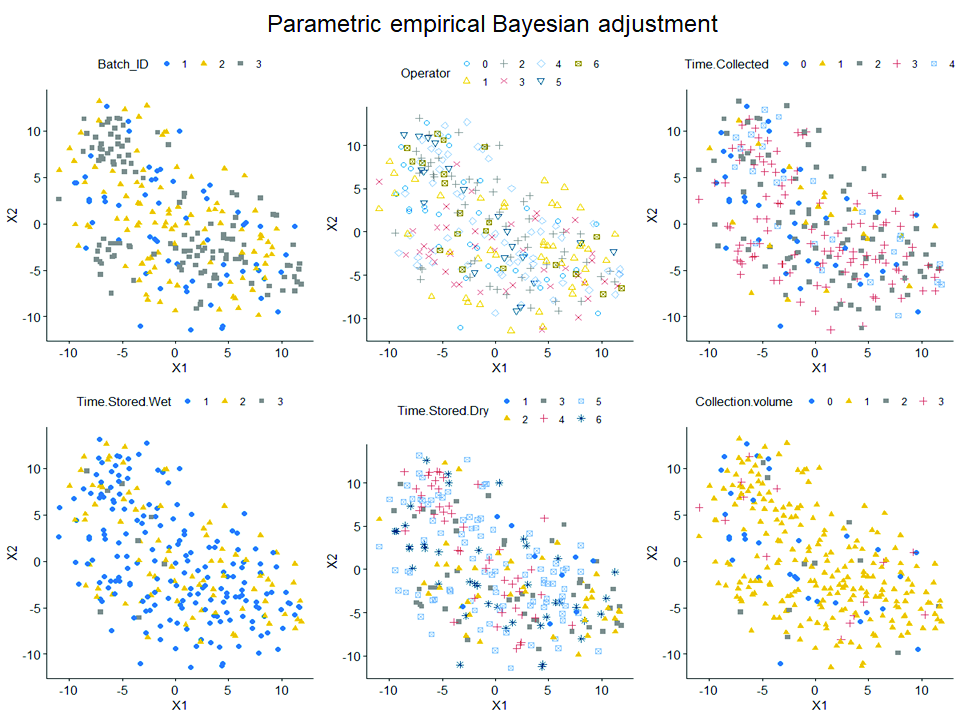


**Figure S4. A 2-dimensional visualization of the high dimensional peak table after adjustment for date of collection ‘Batch_ID’.** Clustering is no longer visible following parametric empirical Bayesian adjustment. The visualization for Figures S3 and S4 were produced using t-distributed stochastic neighbour embedding (t-SNE).

**
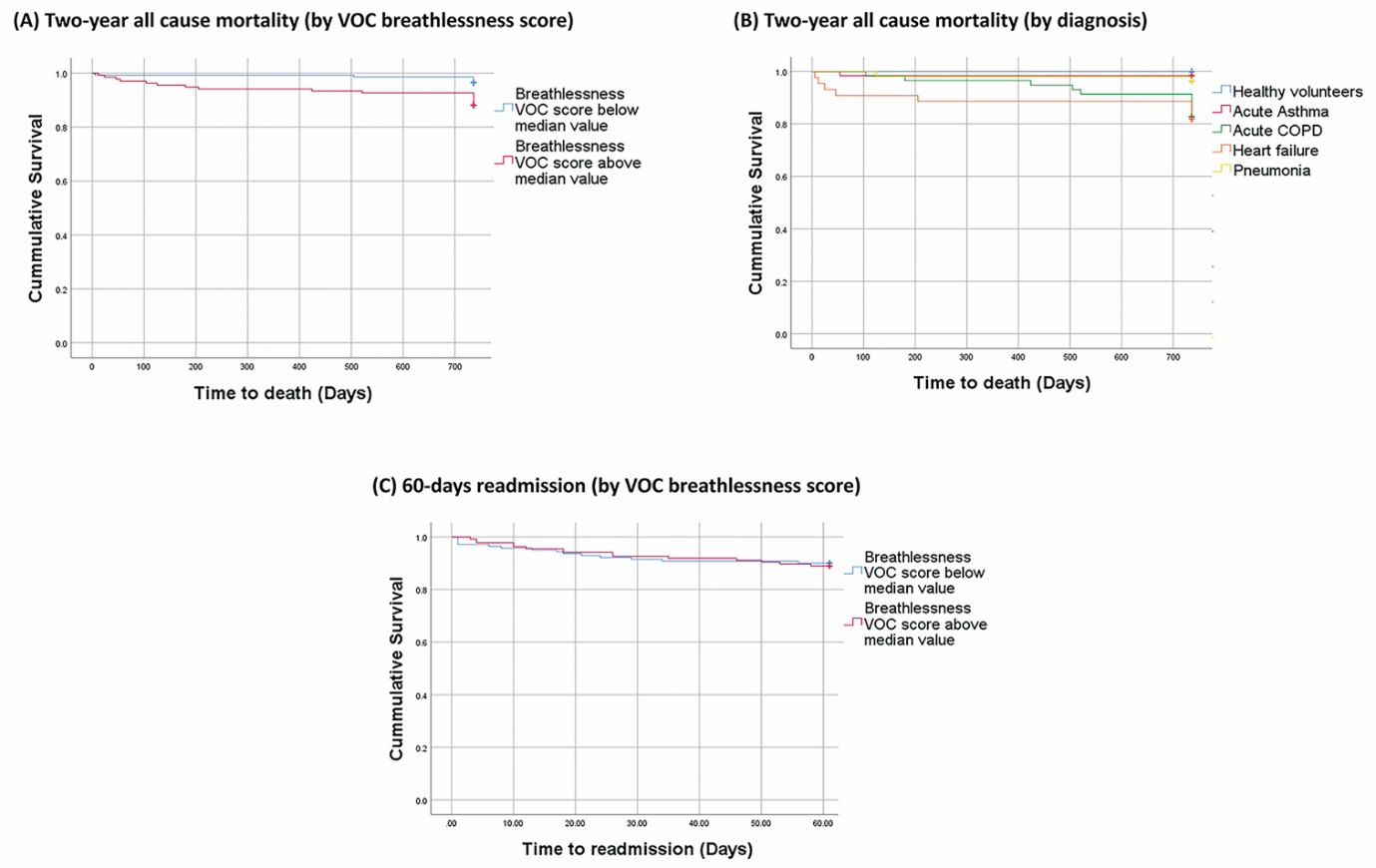
**

**Figure S5. Kaplan-Meier survival analysis.**

**A:** Kaplan-Meier survival analysis for all-cause 2-year mortality, classified by acute disease VOC score median value, showing a significant difference between the groups. P value of 0.009 *(log – rank test (Mantel-Cox) for equality f survivor function).* **B:** Kaplan-Meier survival analysis for all-cause 2-year mortality, classified by disease groups, P value of 0.04 *(log – rank test (Mantel-Cox) for equality f survivor function)*. **C:** Total number of patients readmitted classified by their acute disease VOC score median value, showing no significant difference in the readmission rate based on the underlying VOC score p-value of 0.79 *(log – rank test (Mantel-Cox) for equality f survivor function)*.

**
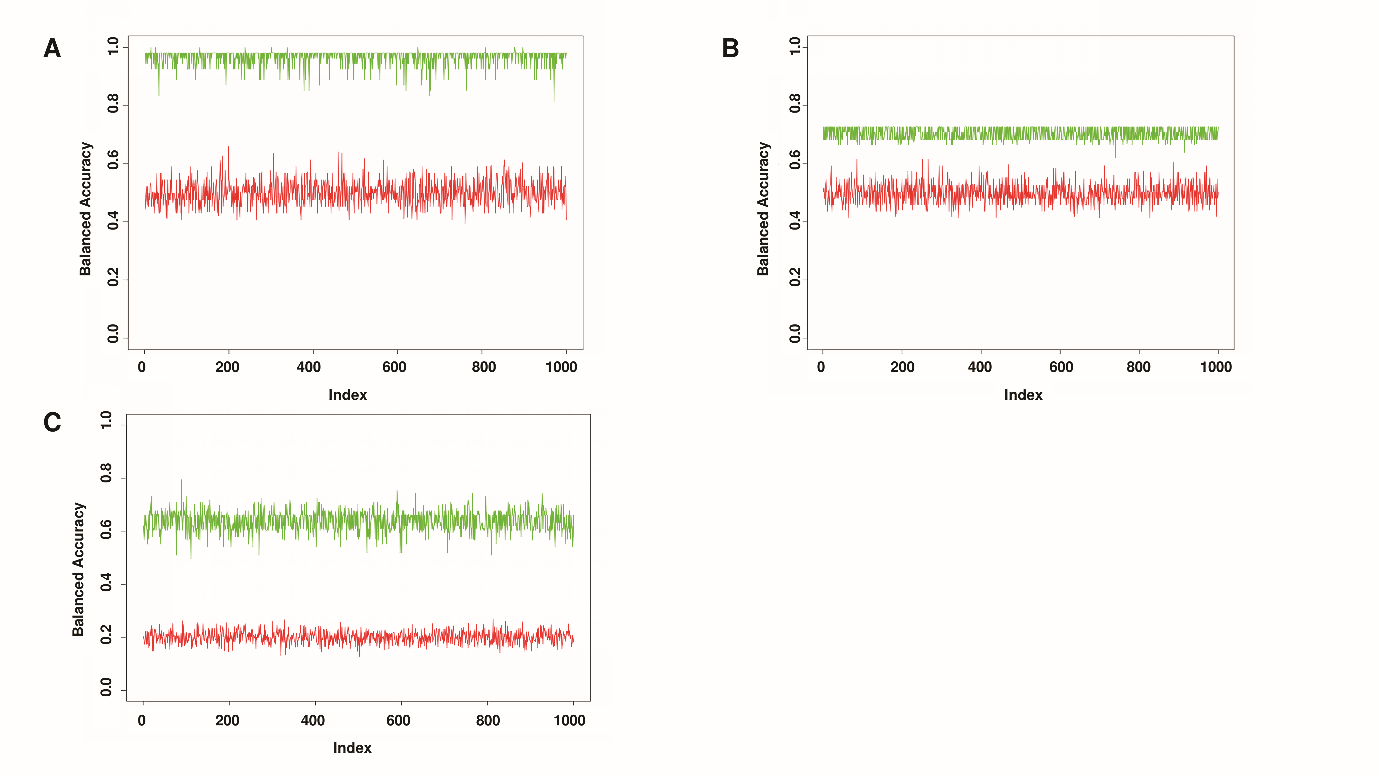
**

**Figure S6. Balanced accuracy predictive performance A:** Predictive performance via the balanced accuracy of model for breathlessness. This model was trained using true class labels (green curve) and balanced accuracy of model for breathlessness trained using randomly shuffled class labels (red curve) in Discovery. Averaged balanced accuracy for model trained using true class labels in Discovery was 96%. The average balanced accuracy for the model trained on the true class labels is significantly different in Discovery (permutation test (x1000 times), p<0.00001) from the average balanced accuracy for the model trained on the randomly shuffled labels. **B:** Predictive performance via the balanced accuracy of model for breathlessness trained using true class labels (green curve) and randomly shuffled class labels (red curve) in Replication. Averaged balanced accuracy for model trained using true class labels in Replication was 75%. The average balanced accuracy for the model trained on the true class labels is significantly different in Replication (permutation test (x1000 times), p<0.00001) from the model trained on the randomly shuffled labels. **C:** The overall classification accuracy for the statistical model using all five biomarker scores from the final set of 101 exhaled breath features was assessed by comparing the balanced accuracy of model trained using the true class labels versus the balanced accuracy of the same model tested using randomly shuffled class labels. This process was repeated 1000 times. The overall classification accuracy using all five biomarker scores was 75%,95% CI (66-77%).

**

**

**Figure S7. Comparative ROC analysis of the diagnostic accuracy of disease VOC score. A:** Comparative ROC analysis demonstrating the diagnostic value of asthma VOC score against the predominantly infection-driven acute disease groups (pneumonia and COPD) in the pooled (discovery and replication) cohorts. **B:** Comparative ROC analysis demonstrating the diagnostic value of heart failure VOC score against other acute disease subgroups (asthma, COPD and pneumonia) in the pooled cohorts.

**

**

**Healthy VOC biomarker score**

**COPD VOC biomarker score**

**Asthma VOC biomarker score**

**Pneumonia VOC biomarker score**

**Heart failure VOC biomarker score**

**Figure S8**. **The difference between VOC biomarker scores.** Violin plots demonstrating significant differences between VOC biomarker scores values across the different disease sub-groups. * Kruskal-Wallis test comparing non-parametric data. * Significant *p-value* <0.0001

**

**

**Figure S9.** **Distribution of the final breath biomarkers across the different disease groups**. Venn diagram demonstrating the final panel of 101 exhaled breath biomarkers and their distribution across disease groups

**
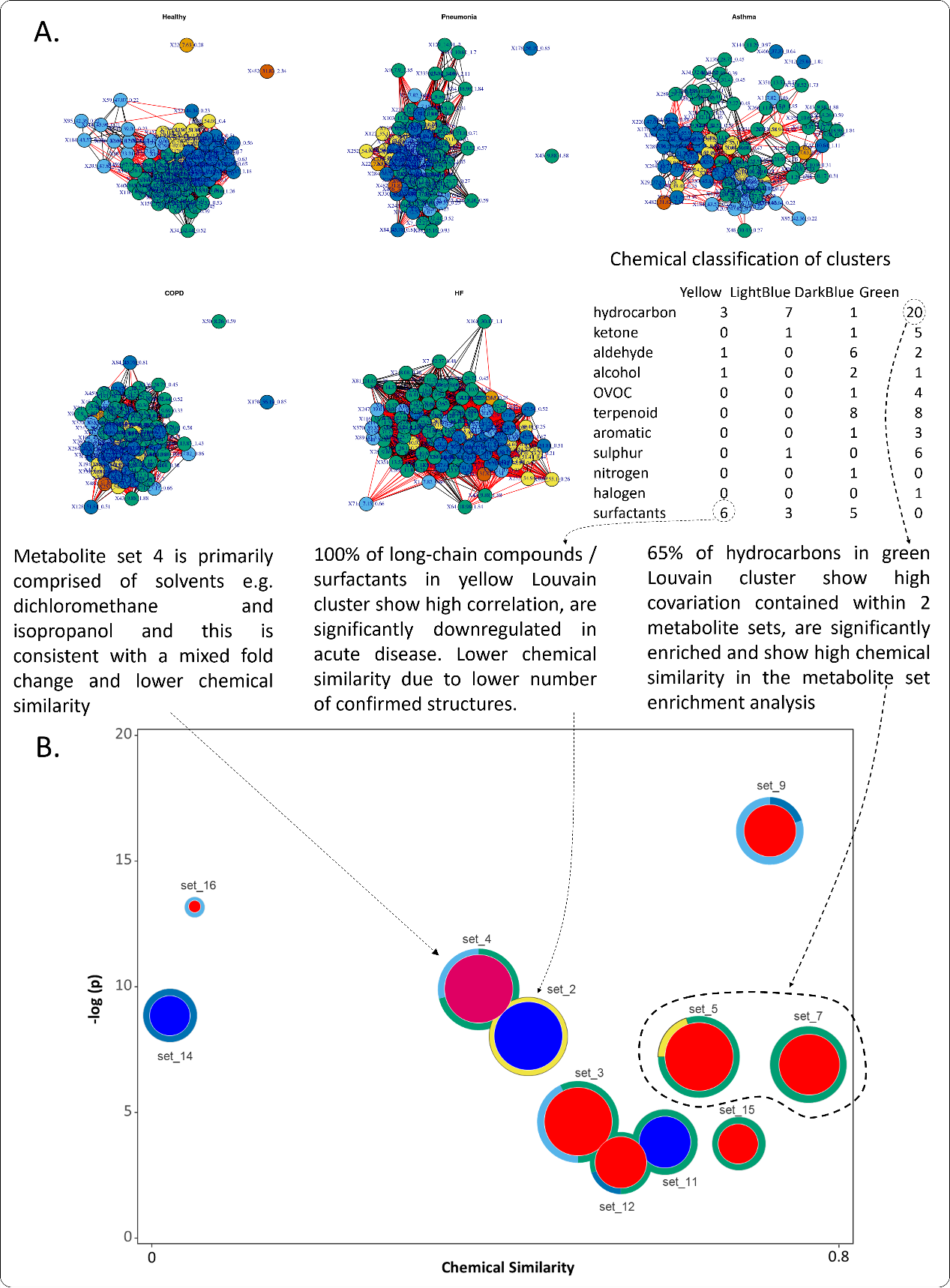
**

**Figure S10. Chemical classification clusters. A**: Correlation graphs showing how the breath metabolites (panel of 101) are correlated within each of the casual subgroups, coloured based on Louvain clusters to highlight differences across the networks. Visual differences highlighted include the green Louvain cluster, being highly compact in the control group and dispersed in the acute groups. **B**: Output of the ChemRICH analysis, showing metabolite sets (circles) significantly enriched during acute breathlessness (size indicative of fold change; red = upregulated; blue =downregulated). The upregulated metabolite sets with high chemical similarity (based on Tanimoto coefficient) consisted predominantly of acyclic and branched hydrocarbons, belonging to the green Louvain cluster (indicated by outer ring colour). The quantitative output of the ChemRICH analysis complements the visual differences in the graph networks.





**Figure S11. 10-fold cross-validation estimates for α at step 4.** Plot shows each of the *λ* (log10 scale) and *α* where z = (cvm - min(cvm)) / sd(cvm). The colour scale is set to have low values (values near the minimum mean cross validation error, cvm) dark green. Values further from the minimum are lighter green, then white, then purple. A red cross identifies the minimum mean cross-validation error. λ is the overall penalty, α is the elastic net penalty.





**Figure S12. 10-fold cross-validation estimates for α at step 5.** Plot shows each of the *λ* (log10 scale) and *α* where z = (cvm - min(cvm)) / sd(cvm). The colour scale is set to have low values (values near the minimum mean cross validation error, cvm) dark green. Values further from the minimum are lighter green, then white, then purple. A red cross identifies the minimum mean cross- validation error. λ is the overall penalty, α is the elastic net penalty.


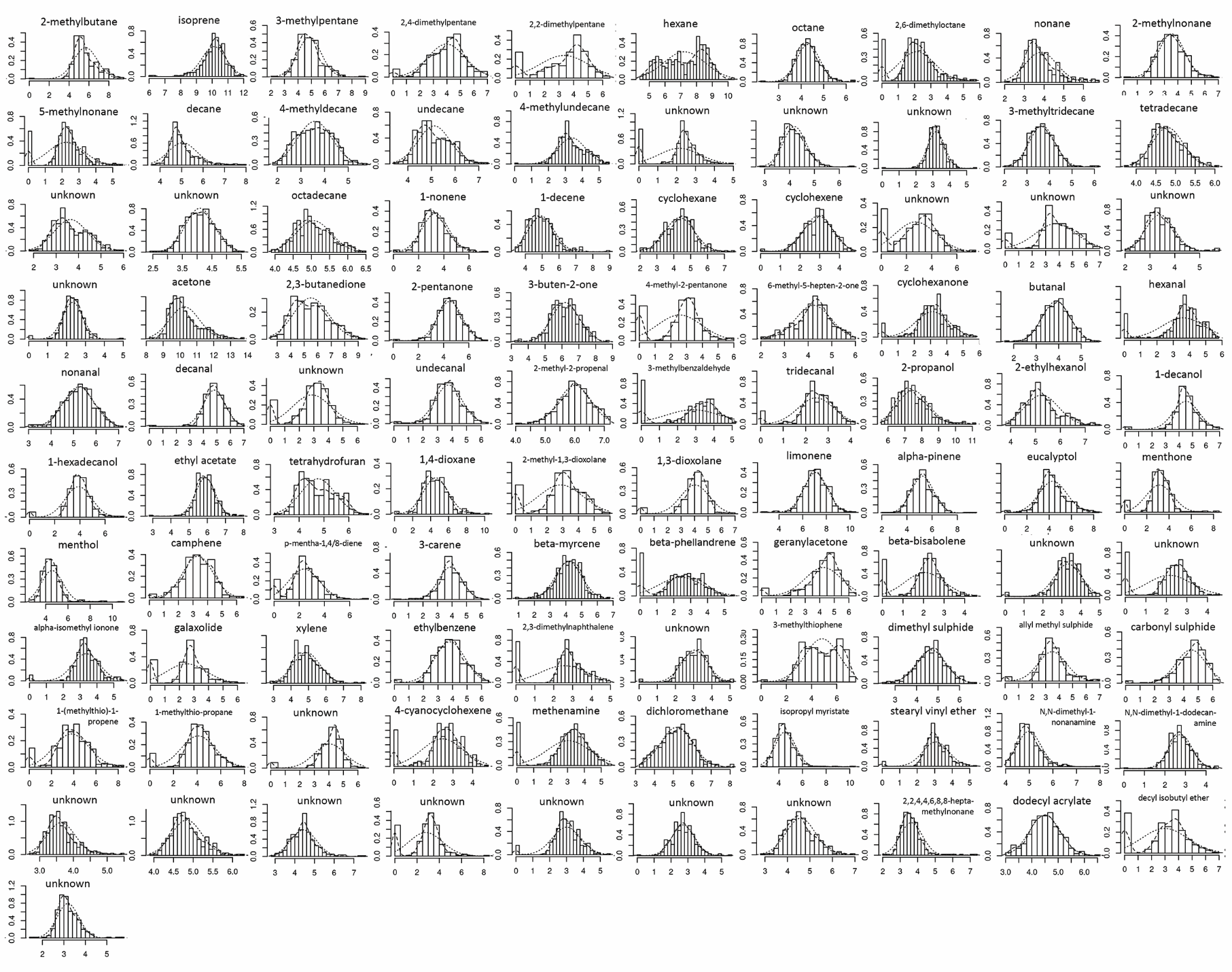


**Figure S13. Graphical probability distribution of the final 101 exhaled breath features in the GCxGC-MS peak data**. The features largely follow a similar distribution. Some features contained a mixture of zero and non-zero values, which have arisen owing to the measurement being below the instrument’s lower limit of detection. Constant features (all zero values) were removed prior to fitting the main model.

**Supplementary Tables:**

|  | **Precision** | **Marginal Error** | **Prevalence of Acute Breathlessness** | **Sample Size Required** |
| --- | --- | --- | --- | --- |
| Sensitivity (70%) | 95% | 10% | 80% | 100 |
| Specificity (70%) | 95% | 10% | 80% | 403 |
| Sensitivity (70%) | 95% | 15% | 80% | 45 |
| Specificity (70%) | 95% | 15% | 80% | 180 |
| Sensitivity (70%) | 95% | 20% | 80% | 25 |
| Specificity (70%) | 95% | 20% | 80% | 100 |
| Sensitivity (80% | 95% | 10% | 80% | 77 |
| Specificity (80%) | 95% | 10% | 80% | 307 |
| **Sensitivity**  **(80%)** | **95%** | **15%** | **80%** | **34** |
| **Specificity (80%** | **95%** | **15%** | **80%** | **137** |
| Sensitivity (80%) | 95% | 20% | 80% | 19 |
| Specificity (80%) | 95% | 20% | 80% | 77 |

**Table S1. Sample size estimation.** Breath biomarker VOC score of acute cardiorespiratory exacerbations demonstrating combined diagnostic accuracy (sensitivity and specificity) of ≥ 80%, with a precision of 95% and maximal marginal error of ±15% in discovery and replication cohorts.

|  | **Discovery** | **Replication** | p value |
| --- | --- | --- | --- |
| Total number (n=) | **139** | **138** |  |
| **Acute asthma** (n=) | 33 | 32 |  |
| **Acute COPD** (n=) | 29 | 29 |  |
| **Pneumonia** (n=) | 28 | 27 |  |
| **Heart failure** (n=) | 22 | 22 |  |
| **Healthy volunteers** (n=) | 27 | 28 |  |
| **Demographics** |  |  |  |
| **Age** (years) mean ± (SD) | 60.6 ± (16.9) | 61.0 ± (16.8) | .846 |
| **Gender** Male (n=) (%) | 65 (46%) | 78 (56%) | .104**¥** |
| **Height** (meters)* | 1.66 ± (0.13) | 1.68 ± (0.16) | .215 |
| **Weight** (kilograms)* | 82.5 ± (21.1) | 85.7± (25.6) | .260 |
| **Body Mass Index** (BMI)* | 29.5 ± (6.7) | 29.6 ± (7.9) | .896 |
| **Breathlessness** |  |  |  |
| **Breathlessness VAS score** (mm)* | 56.1 ± (32.3) | 60.2 ± (30.7) | 0.292 |
| **V1 cough VAS score** (mm)* | 41.6 ± (33.3) | 44.5 ± (33.2) | 0.479 |
| **V1 wheeze VAS score** (mm)* | 40.65± (35.1) | 43.1± (34.8) | 0.558 |
| **Laboratory parameters** |  |  |  |
| **C-Reactive Protein** (mg/dl) | 10.0 (1.0-449.0) | 12.0 (1.0-321.0) | 0.740 |
| **Blood eosinophil count** 10ᶺ9/L | 0.13 (0.01-1.9) | 0.13 (0.01-2.15) | 0.825 |
| **Troponin T** (ng/l) | 3.4 (1.0-1658.4) | 3.15 (1.0-810.1) | 0.565 |
| **BNP** (ng/l) | 40.1 (1.0-1576.0) | 42.6 (1.0-2631.9) | 0.780 |

**Table S2. Baseline demographics and clinical characteristics table.** The table outlines the baseline demographics and clinical characteristics of the discovery and replication cohorts. VAS: Visual Analogue Scale (100mm), participants were asked to rate their breathlessness, cough and wheeze on a 100mm VAS on admission. ANOVA was used to assess the differences between groups for normally distributed continuous variables and Kruskal-Wallis for non-parametric continuous variables. Pearson chi-squared and Fisher’s exact were used to assess the differences in categorical variables. The results were considered statistically significant at *p*-values <0.05. * Data is expressed as mean (SD) or n (%) ± (SD).

| **Comorbidities** | **Healthy controls N (%)** | **Acute disease group N (%)** |
| --- | --- | --- |
| Anxiety/Depression | 5 (9) | 26 (11.7) |
| Diabetes Mellitus | 4 (7.2) | 45 (20) |
| Essential hypertension | 16 (29) | 58 (26) |
| Ischaemic heart disease | 4 (7.2) | 13 (5.8) |
| Arthritis | 5 (9) | 12 (5.4) |
| Thyroid disorder | 3 (5) | 15 (6.7) |
| Chronic Obstructive pulmonary disease | 0 (0) | 58 (26) |
| Asthma | 0 (0) | 65 (29) |
| Heart failure | 0 (0) | 44 (19.8) |
| **Medications** |  |  |
| **Inhaled therapies** |  |  |
| Salbutamol | 1 (1.8) | 153 (68) |
| Inhaled corticosteroids/Long acting B-agonists (ICS/LABA) | 0 (0) | 53 (23) |
| **Lipid lowering agents** |  |  |
| Atorvastatin | 9 (16) | 57 (25.6) |
| Simvastatin | 7 (12) | 23 (10) |
| **Gastroesophageal reflux disease (GORD) medications** |  |  |
| Lansoprazole | 9 (16) | 49 (22) |
| **Blood pressure lowering agents** |  |  |
| Amlodipine | 5 (9) | 25 (11.2) |
| Lisinopril | 4 (7) | 11 (5) |
| Ramipril | 8 (14) | 37 (16.6) |
| **Antidepressants** |  |  |
| Citalopram | 3 (5) | 13 (6) |
| Sertraline | 3 (5) | 15 (6.7) |
| **Thyroid medications** |  |  |
| Levothyroxine | 5 (9) | 15 (6.7) |
| **Cardiac medications** |  |  |
| Aspirin | 5 (9) | 36 (16.2) |
| **Analgesics** |  |  |
| Paracetamol | 6 (10) | 103 (46) |

**Table S3. Comorbidities and medications table.** The table demonstrates comorbidities and medications used by study participants, classified by disease and health. Values expressed as N (%). Table includes comorbidities occurring in >5% of participants and medications used by >5% of participants

| **LASSO on full feature matrix** |  | **LASSO on feature matrix with 7 features removed** |  |
| --- | --- | --- | --- |
| Multinomial Deviance | 3.00 | Multinomial Deviance | 2.93 |
| **Miss-classification Error** | 0..60 | **Miss-classification Error** | 0.59 |
| Mean Squared Error | 0.75 | Mean Squared Error | 0.74 |
| Mean Absolute Error | 1.54 | Mean Absolute Error | 1.52 |

**Table S4. Model performance on full feature matrix and feature matrix with 7 features present**

| **LASSO on full feature**  **matrix** |  | **LASSO on feature matrix with all features present in less**  **than 80% of samples removed** |  |
| --- | --- | --- | --- |
| Multinomial Deviance | 3.00 | Multinomial Deviance | 1.73 |
| **Miss-classification**  **Error** | 0.60 | Miss-classification Error | 0.19 |
| Mean Squared Error | 0.75 | Mean Squared Error | 0.44 |
| Mean Absolute Error | 1.54 | Mean Absolute Error | 1.10 |

**Table S5. Model performance on full feature matrix and feature matrix with all features present in less than 80% of samples removed.**

| **Iteration 1** |  |
| --- | --- |
| Multinomial Deviance | 1.73 |
| **Miss-classification Error** | **0.19** |
| Mean Squared Error | 0.44 |
| Mean Absolute Error | 1.10 |

**Table S6**. **LASSO on feature matrix with all features present in less than 80%** **(iteration 1 of the e-NET feature reduction approach) of samples removed**

| **Iteration 2** |  |
| --- | --- |
| Multinomial Deviance | 1.25 |
| **Miss-classification Error** | **0.10** |
| Mean Squared Error | 0.30 |
| Mean Absolute Error | 0.86 |

**Table S7**. **Elastic net model to the active set from iteration 1 (α =0.5)**

| **Iteration 3** |  |
| --- | --- |
| Multinomial Deviance | 1.12 |
| **Miss-classification Error** | **0.07** |
| Mean Squared Error | 0.26 |
| Mean Absolute Error | 0.80 |

**Table S8. Fit an elastic net model to the active set from iteration 2 (α=0)**

| **Chemical Name** | **CAS-RN** | **KEGG, C- / HMBD, H-** | **ChEBI** | **MSI Level** | **conc. μg/m^3^** | **Log_2_(FC)** | **Acute risk score classification** | **Louvain set** |
| --- | --- | --- | --- | --- | --- | --- | --- | --- |
| *Hydrocarbons* | | | | | | | |  |
| 2-methylbutane | 78-78-4 | - | 30362 | 1 | 0-186 | -0.032 | pneumonia | 3 |
| isoprene | 78-79-5 | C16521 | 35194 | 1 | 7-1,494 | 0.015 | heart failure | 3 |
| 3-methylpentane | 96-14-0 | HMDB0061885 | 88373 | 1 | - | 0.056 | asthma | 3 |
| 2,4-dimethylpentane | 108-08-7 | - | - | 1 | 0-15 | 0.091 | pneumonia | 3 |
| 2,2-dimethylpentane | 590-35-2 | - | - | 2 | - | 0.284† | pneumonia | 3 |
| hexane | 110-54-3 | C11271, HMDB0029600 | 29021 | 1 | 0-781 | 0.083† | asthma, pneumonia, heart failure | 3 |
| octane | 111-65-9 | C01387, HMDB0001485 | 17590 | 1 | 0-2 | -0.030 | pneumonia, COPD | 4 |
| 2,6-dimethyloctane | 2051-30-1 | - | - | 1 | 0-1 | 0.056 | pneumonia | 4 |
| nonane | 111-84-2 | C02445, HMDB0029595 | 32892 | 1 | 0-3 | -0.062 | COPD | 4 |
| 2-methylnonane | 871-83-0 | - | - | 1 | 0-3 | 0.042 | asthma | 4 |
| 5-methylnonane | 15869-85-9 | - | - | 2 | - | 0.102 | heart failure | 4 |
| decane | 124-18-5 | - | 41808 | 1 | 0-8 | 0.017 | asthma | 4 |
| 4-methyldecane | 2847-72-5 | HMDB0037268 | 88816 | 1 | 0-1 | 0.049 | heart failure | 1 |
| undecane | 1120-21-4 | HMDB0031445 | 46342 | 1 | 0-4 | 0.036 | heart failure | 1 |
| 4-methylundecane | 2980-69-0 | - | - | 2 | - | 0.045 | COPD | 1 |
| unknown (branched C12) | - | - | - | 3 | - | 0.277† | COPD | 1 |
| unknown (branched C12) | - | - | - | 3 | - | 0.049† | heart failure, control | 6 |
| unknown (dimethylundecane isomer) | - | - | - | 3 | - | 0.005 | pneumonia | 1 |
| 3-methyltridecane | 6418-41-3 | - | - | 2 | - | 0.102† | control | 6 |
| tetradecane | 629-59-4 | HMDB0059907 | 41253 | 1 | 0-1 | 0.048† | asthma, pneumonia | 6 |
| unknown (branched C14) | - | - | - | 3 | - | 0.103† | pneumonia, control | 6 |
| unknown (branched C14) | - | - | - | 3 | - | 0.042 | pneumonia | 1 |
| unknown (branched C15) | - | - | - | 3 | - | 0.046† | heart failure | 6 |
| octadecane | 593-45-3 | HMDB0033721 | 32926 | 1 | - | -0.061† | control | 7 |
| 1-nonene | 124-11-8 | C08452, HMDB0031270 | 77443 | 1 | 0-4 | -0.050 | asthma | 4 |
| 1-decene | 872-05-9 | - | 87315 | 1 | 0-29 | 0.029 | pneumonia | 4 |
| cyclohexane | 110-82-7 | C11249, HMDB0029597 | 29005 | 1 | 1-9 | 0.132† | COPD, control | 3 |
| cyclohexene | 110-83-8 | - | 36404 | 1 | 0-1 | 0.096 | heart failure | 4 |
| unknown (cyclohexadiene isomer) | - | - | - | 3 | - | 0.639† | control | 5 |
| unknown (methylcyclopentadiene) | - | - | - | 3 | - | 0.416† | COPD, control | 4 |
| unknown (hexadecene isomer) | - | - | - | 3 | - | 0.003 | control | 7 |
| unknown | - | - | - | 3 | - | 0.010 | pneumonia, COPD | 1 |
| *Ketones* | | | | | | | |  |
| acetone | 67-64-1 | C00207, HMDB0001659 | 15347 | 1 | 38-10,077 | 0.062† | heart failure, control | 5 |
| 2,3-butanedione | 431-03-8 | C00741, HMDB0003407 | 16583 | 1 | 0-113 | 0.289† | asthma, COPD | 3 |
| 2-pentanone | 107-87-9 | C01949, HMDB0034235 | 16472 | 1 | 0-6 | 0.106† | asthma | 5 |
| 3-buten-2-one (methyl vinyl ketone) | 78-94-4 | C20701, HMDB0061873 | 48058 | 1 | 0-52 | 0.078† | pneumonia | 3 |
| 4-methyl-2-pentanone | 108-10-1 | C19263, HMDB0002939 | 142806 | 1 | 0-3 | -0.126 | control | 2 |
| 6-methyl-5-hepten-2-one | 110-93-0 | C07287, HMDB0035915 | 16310 | 1 | 0-1 | 0.115† | COPD, control | 4 |
| cyclohexanone | 108-94-1 | C00414, HMDB0003315 | 17854 | 1 | 0-2 | 0.263† | pneumonia, control | 6 |
| *Aldehydes* | | | | | | | |  |
| butanal | 123-72-8 | C01412, HMDB0003543 | 15743 | 1 | - | -0.007 | heart failure | 4 |
| hexanal | 66-25-1 | C02373, HMDB0005994 | 121338 | 1 | 0-1 | 0.002 | asthma, pneumonia | 8 |
| nonanal | 124-19-6 | HMDB0059835 | 84268 | 1 | 0-7 | 0.004 | asthma | 8 |
| decanal | 112-31-2 | C12307, HMDB0011623 | 31457 | 1 | 0-5 | -0.031 | asthma | 8 |
| unknown (methyldecanal isomer) | - | - | - | 3 | - | 0.004 | asthma | 8 |
| undecanal | 112-44-7 | HMDB0030941 | 46202 | 1 | 0-4 | -0.127† | asthma | 8 |
| 2-methyl-2-propenal (methacrolein) | 78-85-3 | HMDB0061874 | 88384 | 1 | 0-2 | 0.016 | pneumonia, heart failure | 3 |
| 3-methylbenzaldehyde | 620-23-5 | C07209, HMDB0029637 | 28476 | 1 | - | 0.107 | asthma | 4 |
| tridecanal | 10486-19-8 | HMDB0030928 | 89816 | 2 | - | -0.108† | heart failure | 7 |
| *Alcohols* | | | | | | | |  |
| 2-propanol | 67-63-0 | C01845, HMDB0000863 | 17824 | 1 | 2-719 | -0.041† | pneumonia, control | 5 |
| 2-ethylhexanol | 104-76-7 | C02498, HMDB0031231 | 16011 | 1 | 0-3 | -0.014 | asthma | 6 |
| 1-decanol | 112-30-1 | C01633, HMDB0011624 | 28903 | 1 | 0-5 | -0.013 | COPD | 8 |
| 1-hexadecanol | 36653-82-4 | C00823, HMDB0003424 | 16125 | 1 | 0-14 | -0.046 | asthma, pneumonia | 7 |
| *Other oxygen-containing VOCs (OVOCs)* | | | | | | | |  |
| ethyl acetate | 141-78-6 | C00849, HMDB0031217 | 27750 | 1 | 1-40 | 0.022 | heart failure | 3 |
| tetrahydrofuran | 109-99-9 | HMDB0000246 | 26911 | 1 | 0-5 | -0.017 | asthma | 4 |
| 1,4-dioxane | 123-91-1 | C14440 | 47032 | 1 | 0-276 | 0.105 | asthma | 3 |
| 2-methyl-1,3-dioxolane | 497-26-7 | - | - | 2 | - | 0.080 | asthma, COPD | 3 |
| 1,3-dioxolane | 646-06-0 | - | 87597 | 2 | - | 0.199† | heart failure | 3 |
| *Terpenes / Terpenoids* | | | | | | | |  |
| limonene | 5989-27-5 | C06099, HMDB0003375 | 15382 | 1 | 0-75 | 0.062† | COPD, heart failure | 2 |
| alpha-pinene | 7785-26-4 | C06308, HMDB0035658 | 28660 | 1 | 0-53 | -0.066 | pneumonia, control | 2 |
| eucalyptol | 470-82-6 | C09844, HMDB0004472 | 27961 | 1 | 0-16 | 0.008 | asthma, COPD | 2 |
| menthone | 14073-97-3 | C00843, HMDB0035162 | 15410 | 1 | 0-5 | -0.097 | pneumonia, COPD | 2 |
| menthol | 2216-51-5 | C00400, HMDB0003352 | 15409 | 1 | 0-160 | 0.023 | COPD | 2 |
| camphene | 79-92-5 | C06076, HMDB0059839 | 3830 | 1 | 0-2 | -0.056 | COPD | 2 |
| p-mentha-1,4/8-diene | 99-85-4 | C09900, HMDB0038150 | 10577 | 2 | - | -0.057 | asthma, pneumonia | 2 |
| 3-carene | 13466-78-9 | C11382, HMDB0035619 | 35661 | 1 | 0-2 | -0.032 | asthma, heart failure | 2 |
| beta myrcene | 123-35-3 | C06074, HMDB0038169 | 17221 | 1 | 0-2 | -0.002 | heart failure | 2 |
| beta-phellandrene | 555-10-2 | C19818, HMDB0036081 | 48741 | 2 | - | 0.008 | asthma, pneumonia | 2 |
| geranylacetone | 3796-70-1 | C13297, HMDB0031846 | 67206 | 1 | 1-6 | 0.177† | control | 8 |
| beta-bisabolene | 495-61-4 | C16775, HMDB0035992 | 49249 | 2 | - | -0.198 | asthma | 7 |
| unknown (sesquiterpenoid) | - | - | - | 3 | - | -0.082 | asthma, pneumonia | 7 |
| unknown | - | - | - | 3 | - | 0.231 | COPD | 8 |
| alpha isomethyl ionone | - | - | - | 2 | - | -0.030 | control | 7 |
| galaxolide | 1222-05-5 | - | 83784 | 2 | - | -0.113 | COPD | 7 |
| *Aromatics* | | | | | | | |  |
| xylene | 106-42-3 | C06756, HMDB0059924 | 27417 | 1 | 0-5 | 0.12† | asthma, pneumonia, control | 4 |
| ethylbenzene | 100-41-4 | C07111, HMDB0059905 | 16101 | 1 | 0-1 | 0.15† | heart failure | 4 |
| 2,3-dimethylnaphthalene | 581-40-8 | - | 48615 | 1 | - | -0.05 | COPD, heart failure | 7 |
| unknown (C9, substituted benzene) | - | - | - | 3 | - | 0.235† | control | 4 |
| *Sulphur-containing VOCs* | | | | | | | |  |
| 3-methyl thiophene | 616-44-4 | HMDB0033119 | 89007 | 1 | 0-7 | 0.040 | COPD | 3 |
| dimethyl sulphide | 75-18-3 | C00580, HMDB0002303 | 17437 | 1 | 0-16 | -0.044 | control | 3 |
| allyl methyl sulphide | 10152-76-8 | HMDB0031653 | 89856 | 1 | 0-4 | -0.230† | COPD, control | 2 |
| carbonyl sulphide | 463-58-1 | C07331 | 16573 | 2 | - | 0.122† | pneumonia, COPD | 3 |
| 1-(methylthio)-1-propene | 10152-77-9 | HMDB0059843 | 89721 | 1 | 0-1,126 | -0.186 | pneumonia | 2 |
| 1-methylthio-propane | 3877-15-4 | HMDB0061871 | 88383 | 2 | - | -0.044 | pneumonia | 2 |
| unknown (C4 thio-containing) | - | - | - | 3 | - | -0.007 | asthma | 2 |
| *Nitrogen-containing VOCs* | | | | | | | |  |
| 4-cyanocyclohexene | 100-45-8 | - | - | 1 | - | -0.039 | asthma, pneumonia | 7 |
| methenamine | 100-97-0 | D00393,HMDB0029598 | 6824 | 2 | - | 0.014 | asthma, pneumonia | 7 |
| *Halogenates* | | | | | | | |  |
| dichloromethane | 75-09-2 | C02271, HMDB0031548 | 15767 | 1 | 0-199 | -0.007 | pneumonia, COPD | 5 |
| *Surfactants and emollients* | | | | | | | |  |
| isopropyl myristate | 110-27-0 | D02296,HMDB0040392 | 90027 | 1 | 0-76 | -0.189† | control | 7 |
| stearyl vinyl ether | 930-02-9 | - | - | 2 | - | -0.235† | asthma, control | 7 |
| N,N-dimethyl-1-nonanamine | 17373-27-2 | - | - | 2 | - | 0.055† | asthma, heart failure | 6 |
| N,N-dimethyl-1-dodecanamine | 112-18-5 | - | - | 2 | - | 0.056 | COPD | 6 |
| unknown (alkenyl hexanoic acid ester) | - | - | - | 3 | - | -0.061 | control | 7 |
| unknown (alkenyl hexanoic acid ester) | - | - | - | 3 | - | -0.042 | control | 7 |
| unknown (alkenyl hexanoic acid ester) | - | - | - | 3 | - | -0.038 | COPD, heart failure | 7 |
| unknown (surfactant) | - | - | - | 3 | - | 0.134 | asthma | 8 |
| unknown (emoillent) | - | - | - | 3 | - | -0.162 | asthma | 7 |
| unknown (eicosanol) | - | - | - | 3 | - | -0.165† | control | 6 |
| unknown (emoillent) | - | - | - | 3 | - | -0.013 | asthma | 7 |
| 2,2,4,4,6,8,8-heptamethylnonane | 4390-04-9 | - | 131383 | 2 | - | 0.045† | control | 6 |
| dodecyl acrylate | 2156-97-0 | - | - | 2 | - | -0.021 | pneumonia | 7 |
| decyl isobutyl ether | - | - | - | 2 | - | 0.002 | heart failure | 8 |

**Table S9. Chemical assignment table**. The table outlines the assignment of selected predictive markers from the regression model detailing chemical name, CAS registry number, KEGG, Human Metabolome Database and ChEBI identifiers and MSI-compliant metabolite identification level, concentration range and fold change (expressed as log2) between acute and control groups, and compound contribution towards disease-specific biomarker risk scores (†adjusted p-value <0.05).

|  | **logFC** | **AveExpr** | **t** | **P.Value** | **adj.P.Val** | **B** |
| --- | --- | --- | --- | --- | --- | --- |
| **Set3** | **0.133890616** | **0.004263125** | **2.879346419** | **0.004319299** | **0.034554392** | **-2.142608513** |
| **Set1** | -0.120196744 | 0.011684816 | -1.854952871 | 0.064743701 | 0.258974804 | -4.369194843 |
| **Set7** | 0.061793373 | 0.015703055 | 1.157435072 | 0.248165085 | 0.544802368 | -5.347558772 |
| **Set5** | -0.060241661 | -0.027387588 | -0.996694751 | 0.31984657 | 0.544802368 | -5.50959215 |
| **Set6** | -0.044261401 | 0.018231244 | -0.830155612 | 0.407218311 | 0.544802368 | -5.652155219 |
| **Set2** | 0.042555754 | 0.007308014 | 0.827706348 | 0.408601776 | 0.544802368 | -5.65405913 |
| **Set4** | -0.035370385 | 0.012747522 | -0.700755726 | 0.484086974 | 0.553242256 | -5.74507753 |
| **Set8** | 0.007185736 | 0.005059851 | 0.128594967 | 0.897778335 | 0.897778335 | -5.967968995 |

**Table S10. Asthma enrichment analysis.** The table demonstrates the results of the enrichment analysis performed in the asthma group using the 8 feature sets obtained from the Louvain clustering on the correlation graph **(fig. S10).**

|  | **logFC** | **AveExpr** | **t** | **P.Value** | **adj.P.Val** | **B** |
| --- | --- | --- | --- | --- | --- | --- |
| **Set3** | -0.090944102 | 0.004263125 | -1.847462511 | 0.065824888 | 0.31930245 | -4.015728578 |
| **Set5** | -0.101063794 | -0.027387588 | -1.579494626 | 0.115447869 | 0.31930245 | -4.359177814 |
| **Set6** | 0.081933094 | 0.018231244 | 1.451613602 | 0.147823971 | 0.31930245 | -4.504601143 |
| **Set1** | 0.096742783 | 0.011684816 | 1.410314835 | 0.159651225 | 0.31930245 | -4.548997592 |
| **Set4** | 0.058154903 | 0.012747522 | 1.08835486 | 0.277454107 | 0.443926572 | -4.851845137 |
| **Set2** | -0.043701593 | 0.007308014 | -0.802920566 | 0.422759696 | 0.489518896 | -5.055725798 |
| **Set7** | 0.044836139 | 0.015703055 | 0.793305124 | 0.428329034 | 0.489518896 | -5.061530212 |
| **Set8** | 0.002536665 | 0.005059851 | 0.042881813 | 0.965828915 | 0.965828915 | -5.299201629 |

**Table S11. COPD enrichment analysis.** Feature enrichment in COPD using 8 features sets obtained by Louvain clustering on the correlation graph

|  | **logFC** | **AveExpr** | **t** | **P.Value** | **adj.P.Val** | **B** |
| --- | --- | --- | --- | --- | --- | --- |
| **Set3** | **-0.16062091** | **0.004263125** | **-2.841944019** | **0.004842214** | **0.032792407** | **-2.229765975** |
| **Set5** | **0.195734539** | **-0.027387588** | **2.664418025** | **0.008198102** | **0.032792407** | **-2.6741005** |
| **Set2** | 0.147456886 | 0.007308014 | 2.359677827 | 0.019036076 | 0.050762869 | -3.37356668 |
| **Set1** | 0.087558967 | 0.011684816 | 1.111758525 | 0.267276909 | 0.534553819 | -5.37643629 |
| **Set6** | -0.054112112 | 0.018231244 | -0.835023125 | 0.404477256 | 0.64716361 | -5.628009236 |
| **Set7** | -0.040272554 | 0.015703055 | -0.620631141 | 0.535390206 | 0.711290217 | -5.773994793 |
| **Set4** | 0.028289272 | 0.012747522 | 0.461124606 | 0.645097706 | 0.711290217 | -5.85478987 |
| **Set8** | -0.025165368 | 0.005059851 | -0.370531932 | 0.711290217 | 0.711290217 | -5.890086764 |

**Table S12. Heart failure enrichment analysis.** Feature enrichment in heart failure using 8 features sets obtained by Louvain clustering on the correlation graph.

|  | **logFC** | **AveExpr** | **t** | **P.Value** | **adj.P.Val** | **B** |
| --- | --- | --- | --- | --- | --- | --- |
| **Set2** | -0.092675555 | 0.007308014 | -1.658089062 | 0.098514794 | 0.350018518 | -4.257598246 |
| **Set3** | 0.083374576 | 0.004263125 | 1.6493092 | 0.100301314 | 0.350018518 | -4.268325935 |
| **Set6** | 0.082784378 | 0.018231244 | 1.428260455 | 0.154426336 | 0.350018518 | -4.520076707 |
| **Set5** | -0.08936284 | -0.027387588 | -1.36002492 | 0.175009259 | 0.350018518 | -4.590632855 |
| **Set8** | 0.029388432 | 0.005059851 | 0.483786536 | 0.628947737 | 0.86559581 | -5.192394684 |
| **Set7** | -0.024708996 | 0.015703055 | -0.425730243 | 0.670659348 | 0.86559581 | -5.212141706 |
| **Set1** | 0.017148026 | 0.011684816 | 0.243432731 | 0.807863636 | 0.86559581 | -5.257780971 |
| **Set4** | 0.009296591 | 0.012747522 | 0.169424129 | 0.86559581 | 0.86559581 | -5.269216827 |

**Table S13. Pneumonia enrichment analysis.** Feature enrichment in Pneumonia using 8 features sets obtained by Louvain clustering on the correlation graph.

| **Section & Topic** | **No** | **Item** | **Reported on**  **page #** |
| --- | --- | --- | --- |
| **TITLE OR**  **ABSTRACT** |  |  | 1 |
|  | **1** | Identification as a study of diagnostic accuracy using at least one measure of accuracy  (such as sensitivity, specificity, predictive values, or AUC) | 2 |
| **ABSTRACT** |  |  |  |
|  | **2** | Structured summary of study design, methods, results, and conclusions  (for specific guidance, see STARD for Abstracts) | 2 |
| **INTRODUCTION** |  |  |  |
|  | **3** | Scientific and clinical background, including the intended use and  clinical role of the index test | 3 |
|  | **4** | Study objectives and hypotheses | Supplementary material – section 1 |
| **METHODS** |  |  |  |
| *Study design* | **5** | Whether data collection was planned before the index test and reference standard  were performed (prospective study) or after (retrospective study) | 10 |
| *Participants* | **6** | Eligibility criteria | Supplementary material |
|  | **7** | On what basis potentially eligible participants were identified  (such as symptoms, results from previous tests, inclusion in registry) | 10 and Supplementary material |
|  | **8** | Where and when potentially eligible participants were identified  (setting, location and dates) | 10 |
|  | **9** | Whether participants formed a consecutive, random or  convenience series | 10 |
| *Test methods* | **10a** | Index test, in sufficient detail to allow replication | 11 |
|  | **10b** | Reference standard, in sufficient detail to allow replication | 11-14 |
|  | **11** | Rationale for choosing the reference standard (if alternatives exist) | 10 |
|  | **12a** | Definition of and rationale for test positivity cut-offs or result categories  of the index test, distinguishing pre-specified from exploratory | 14 |
|  | **12b** | Definition of and rationale for test positivity cut-offs or result categories  of the reference standard, distinguishing pre-specified from  exploratory | 14 |
|  | **13a** | Whether clinical information and reference standard results were available  to the performers/readers of the index test | 10-11 |
|  | **13b** | Whether clinical information and index test results were available  to the assessors of the reference standard | 10-11 |
| *Analysis* | **14** | Methods for estimating or comparing measures of diagnostic  accuracy | 14-15 and supplementary material |
|  | **15** | How indeterminate index test or reference standard results were  handled | Supplementary material |
|  | **16** | How missing data on the index test and reference standard were  handled | 12 |
|  | **17** | Any analyses of variability in diagnostic accuracy, distinguishing pre-  specified from exploratory | Supplementary material |
|  | **18** | Intended sample size and how it was determined | 13 and Supplementary material |
| **RESULTS** |  |  |  |
| *Participants* | **19** | Flow of participants, using a diagram | Supplementary material – Figure S1 |
|  | **20** | Baseline demographic and clinical characteristics of participants | 10 and table 1 |
|  | **21a** | Distribution of severity of disease in those with the target condition | 10 and Supplementary material table S2 –S3 |
|  | **21b** | Distribution of alternative diagnoses in those without the target  condition | 10 and Supplementary material table S3 |
|  | **22** | Time interval and any clinical interventions between index test and reference standard | 10 |
| *Test results* | **23** | Cross tabulation of the index test results (or their distribution)  by the results of the reference standard | 11-14 and Supplementary material |
|  | **24** | Estimates of diagnostic accuracy and their precision (such as 95%  confidence intervals) | 12 and Supplementary material |
|  | **25** | Any adverse events from performing the index test or the reference standard | 11 |
| **DISCUSSION** |  |  |  |
|  | **26** | Study limitations, including sources of potential bias, statistical  uncertainty, and generalisability | 8-9 |
|  | **27** | Implications for practice, including the intended use and clinical role of the index test | 10-11 |
| **OTHER**  **INFORMATION** |  |  |  |
|  | **28** | Registration number and name of registry | 20 |
|  | **29** | Where the full study protocol can be accessed | 10 |
|  | **30** | Sources of funding and other support; role of funders | 20 |

**Table S14. Standards for Reporting Diagnostic accuracy studies (STARD) checklist.**

| **Section/Topic** | **Item** | **Checklist Item** | **Page** |
| --- | --- | --- | --- |
| **Title and abstract** | | | |
| Title | 1 | Identify the study as developing and/or validating a multivariable prediction model, the target population, and the outcome to be predicted. | 1 |
| Abstract | 2 | Provide a summary of objectives, study design, setting, participants, sample size, predictors, outcome, statistical analysis, results, and conclusions. | 2 |
| **Introduction** | | | |
| Background and objectives | 3a | Explain the medical context (including whether diagnostic or prognostic) and rationale for developing or validating the multivariable prediction model, including references to existing models. | 3 |
|  | 3b | Specify the objectives, including whether the study describes the development or validation of the model or both. | Supplementary material |
| **Methods** | | | |
| Source of data | 4a | Describe the study design or source of data (e.g., randomized trial, cohort, or registry data), separately for the development and validation data sets, if applicable. | 10 |
|  | 4b | Specify the key study dates, including start of accrual; end of accrual; and, if applicable, end of follow-up. | 10 |
| Participants | 5a | Specify key elements of the study setting (e.g., primary care, secondary care, general population) including number and location of centres. | 10 |
|  | 5b | Describe eligibility criteria for participants. | Supplementary material |
|  | 5c | Give details of treatments received, if relevant. | N/A |
| Outcome | 6a | Clearly define the outcome that is predicted by the prediction model, including how and when assessed. | 5 |
|  | 6b | Report any actions to blind assessment of the outcome to be predicted. | Supplementary material |
| Predictors | 7a | Clearly define all predictors used in developing or validating the multivariable prediction model, including how and when they were measured. | 5-7 |
|  | 7b | Report any actions to blind assessment of predictors for the outcome and other predictors. | Supplementary material |
| Sample size | 8 | Explain how the study size was arrived at. | 13 and Supplementary material |
| Missing data | 9 | Describe how missing data were handled (e.g., complete-case analysis, single imputation, multiple imputation) with details of any imputation method. | 12 |
| Statistical analysis methods | 10a | Describe how predictors were handled in the analyses. | 5 and Supplementary material |
|  | 10b | Specify type of model, all model-building procedures (including any predictor selection), and method for internal validation. | Supplementary  material |
|  | 10d | Specify all measures used to assess model performance and, if relevant, to compare multiple models. | Supplementary material – section 3.2 |
| Risk groups | 11 | Provide details on how risk groups were created, if done. | N/A |
| **Results** | | | |
| Participants | 13a | Describe the flow of participants through the study, including the number of participants with and without the outcome and, if applicable, a summary of the follow-up time. A diagram may be helpful. | Supplementary material – figure S1 |
|  | 13b | Describe the characteristics of the participants (basic demographics, clinical features, available predictors), including the number of participants with missing data for predictors and outcome. | Table 1 |
| Model development | 14a | Specify the number of participants and outcome events in each analysis. | Supplementary  material  Figure S2 |
|  | 14b | If done, report the unadjusted association between each candidate predictor and outcome. | N/A |
| Model specification | 15a | Present the full prediction model to allow predictions for individuals (i.e., all regression coefficients, and model intercept or baseline survival at a given time point). | Supplementary  material |
|  | 15b | Explain how to the use the prediction model. | 12-13 and  Supplementary  material |
| Model performance | 16 | Report performance measures (with CIs) for the prediction model. | Supplementary material – section 3 |
| **Discussion** | | | |
| Limitations | 18 | Discuss any limitations of the study (such as nonrepresentative sample, few events per predictor, missing data). | 8-9 |
| Interpretation | 19b | Give an overall interpretation of the results, considering objectives, limitations, and results from similar studies, and other relevant evidence. | 4-8 |
| Implications | 20 | Discuss the potential clinical use of the model and implications for future research. | 8-9 |
| **Other information** | | | |
| Supplementary information | 21 | Provide information about the availability of supplementary resources, such as study protocol, Web calculator, and data sets. | 15 and Supplementary material |
| Funding | 22 | Give the source of funding and the role of the funders for the present study. | 20 |

**Table S15. Transparent Reporting of multivariate prediction model for Individual Prognosis or Diagnosis (TRIPOD) checklist**
